# Altered value-based decision-making in anorexia nervosa: A systematic review

**DOI:** 10.1016/j.neubiorev.2024.105944

**Published:** 2024-11-17

**Authors:** Carina S. Brown, Audrey Nuñez, Christina E. Wierenga

**Affiliations:** a Department of Psychiatry, University of California, San Diego, USA; b San Diego State University/University of California, San Diego Joint Doctoral Program in Clinical Psychology, USA

**Keywords:** Anorexia nervosa, Decision-making, Executive functioning, Eating disorders, Choice behavior, Valuation

## Abstract

Alterations in decision-making are considered core to anorexia nervosa (AN) phenomenology and may maintain illness through maladaptive choice behavior. This systematic review (*n* = 77) aimed to extend prior reviews beyond standard neuropsychological batteries by incorporating novel value-based choice tasks and computational methods. We organize findings across key factors, including: 1) illness state, 2) developmental stage, and 3) AN subtype, and highlight available neuroimaging findings. Differences in decision-making appear consistent during illness, including in weight-restored samples, but not in recovery and not in all domains. Differences are not consistently present in adolescence, although punishment sensitivity may be heightened; AN subtypes are not consistently distinguishable. Overall, decision-making varies by context and is influenced by reward/punishment processing, risk/uncertainty, and flexibility/control. Utilization of computational modeling methods, possibly increasing precision, highlight that, although raw behavior may not differ at recovery, latent decision-making processes appear impacted. Clinical interventions may benefit from consideration of context when working to shape choice behavior and from consideration of latent decision-making processes that influence how choices are made.

## 1. Introduction

Anorexia nervosa (AN) is a debilitating and serious eating disorder, with one of the highest mortality rates of any psychiatric condition (Arcelus et al., 2011). Characterized by persistent restriction of food intake, an intense fear of gaining weight, and a distorted perception of body weight or shape, it is marked by substantial chronicity for over half of those afflicted (Fichter et al., 2017). Cognitive neuroscience models of AN propose that altered value-based decision-making may contribute to the persistence of disordered eating behavior (Steinglass et al., 2019; Wierenga, 2023). Specifically, difficulty adapting behavior to the environment and to shifting environmental contingencies (e.g., continuing to restrict food despite dangerous weight loss) suggests broad alterations in some executive functions, particularly decision-making.

From a cognitive science perspective, decision-making is a multifaceted set of cognitive processes cumulatively linked to choice behavior (Gonzalez, 2017). Although associated with broader constructs, such as attention and memory, decision-making is inherently influenced by stimulus valuation and action selection prior to choice, execution during choice, and learning following choice to align behavior with short-term or long-term goals (Paulus, 2007). Thus, a decision-maker evaluates options through perception of the current environment to make a choice, and, based on the outcome, can learn whether that decision was consistent with their goal. This process also involves delay of gratification, exploration of new options versus exploitation of gained knowledge, appraisal of reward and threat, and identification of appropriate behaviors, given the current state. Longstanding theories of decision-making, such as expected utility theory (Bernoulli, 1954) and prospect theory (Kahneman and Tversky, 1979), were designed to provide a comprehensive understanding of the cognitive and perceptual mechanisms accompanying valuation of choice and choice behavior, particularly under risk.

Expected utility theory, for example, emphasizes both the absolute value of a choice outcome, as well as the subjective value (i.e., utility), and predicts that individuals will attempt to maximize the amount of utility received. Meanwhile, prospect theory was developed from expected utility theory to address limitations associated with framing effects. Kahneman & Tversky proposed that decision-making consists of editing and evaluation phases; the former organizes and codes possible options and the latter assesses those options and selects a choice. Thus, the decision-maker more flexibly adjusts expected utility based on their understanding and judgement of the choices available to them.

Importantly, the highlighted decision-making models operationalize the process of choice, including mechanisms prior to and following choice behavior, which predict not only what decision will be made, but also how decision-making occurs (Mishra, 2014). While these models and others, including risk sensitivity theory, heuristic approaches, and mean-variance frameworks, are constrained in their ability to fully explain human behavior, given that decision-making in humans can be irrational, dynamic, and influenced by competing processes (e.g., motivation, reward salience; Gonzalez, 2017), they provide important frameworks from which we can begin to understand decision-making in the context of pathological behavior; for example, choices, such as deciding what and what not to eat, can play a critical role in the maintenance of disease.

Efforts to estimate and measure the expected value or reward value of stimuli and choices (e.g., food choices), in Pavlovian and instrumental learning contexts, have led to the identification of neural regions implicated in valuation, specifically the medial orbitofrontal cortex (mOFC) and the ventromedial prefrontal cortex (vmPFC). Both are believed to track expectations in both gain and loss conditions (O’Doherty and Bossaerts, 2008; Rushworth et al., 2011), with prior work showing that vmPFC and mOFC signal increases in tandem with increasing subjective value of food stimuli in healthy participants (Plassmann et al., 2007) and is positively correlated with cue or action valuation as estimated using reinforcement learning models (Doya et al., 2004; Gläscher et al., 2009; Wunderlich et al., 2010). Lateral prefrontal regions, including dorsolateral prefrontal cortex (dlPFC), are also hypothesized to have involvement in representing value-based information, as well as involvement in top-down cognitive control (for a review, see: Dixon and Christoff, 2014). In fact, prior work has identified both dlPFC and vmPFC as key regions involved in cognitive regulation (e.g., value modulation) of food choices in healthy participants, where downregulation of food craving was associated with attenuated activity in dlPFC and upregulation of food craving was associated with augmented activity in vmPFC, reflecting disparate neural mechanisms involved in value computation and its regulation (Hutcherson et al., 2012).

Beyond the process of valuation, choices are also invariably impacted by action selection processes, including exploration-exploitation tradeoffs. Exploration engages control, salience, and attentional networks, particularly the lateral frontopolar cortex (Badre et al., 2012; Daw et al., 2006; Hogeveen et al., 2022), while exploitation primarily recruits the vmPFC and OFC (Hogeveen et al., 2022; Wyatt et al., 2024). Goal-directed and habitual systems, governed by distinct striatal functions, are also believed to contribute to action selection and choice behavior. The ventral-medial striatum is implicated in goal-directed learning, whereas the dorsal striatum is involved in habit learning, with a transition from ventral-medial to dorsal activity reflecting a shift from goal-directed to habitual control (Burton et al., 2015).

Importantly, activity in neural regions involved in these processes have been shown to be altered in individuals with AN; for example, dysfunction in dorsal executive-function neurocircuitry (e.g., dlPFC, dorsal caudate) has been identified as a putative mechanism contributing to imbalances in information processing and cognitive control, which is likely relevant for decision-making in this population (Kaye et al., 2009, 2013). Additionally, studies examining reward anticipation and receipt in individuals with AN have shown reduced activity in reward regions (e.g., ventral striatum, ventral tegmental area; Fladung et al., 2010; Jiang et al., 2019) and increased activity of cognitive control regions (e.g., dlPFC; Ehrlich et al., 2015; Uher et al., 2003, 2004), leading to hypotheses about enhanced top-down control (e.g., altered action selection processes) and possible modulation of reward value (e.g., altered valuation) that results in the ability to ignore palatable food or continuously opt for low-fat foods. Focus on disruptions in dorsal frontostriatal neurocircuitry in particular has led to models of disease progression in AN that hypothesize engagement of reward circuitry (e.g., ventral striatum, vmPFC) at the onset of the disorder and a gradual transition to habit circuitry (e.g., dorsal striatum, dlPFC), mirroring neural representations of shifts in goal-directed to habitual control that could impact disorder-relevant decision-making, resulting in persistent goal pursuit and rigidity of choice behavior (Uniacke et al., 2018).

The role of decision-making in the emergence and maintenance of disorders marked by inflexible goal pursuit has been highlighted extensively, with recent evidence that decision-making differences may precede the onset of an eating disorder (Harrison et al., 2022b, 2022a). Although decision-making, and consequently the ability to obtain objectives, has been shown to be largely impaired across many psychiatric conditions (Griffiths et al., 2014), more recent conceptualizations of this process in eating disorders propose that AN reflects excessive goal pursuit and, in some cases, augmented or improved decision-making (Haynos et al., 2022). Yet, studies continue to find state-related deficits that improve with recovery (Fuglset, 2019; Guillaume et al., 2015). Such discrepancies are further complicated by challenges integrating and understanding decision-making in various contexts (e.g., reward/punishment, uncertainty/risk, and inflexibility/control). For example, while individuals with AN may demonstrate deficits in probabilistic monetary reward tasks (Wierenga et al., 2021), they may also show increased ability in delay discounting tasks (Steward et al., 2017).

### 1.1. Present review

Several systematic reviews have been published in recent years documenting the role of decision-making in the phenomenology of AN (for a review of reviews, see: Smith et al., 2018). However, there remain several gaps in the literature that will be addressed with this review. First, prior reviews largely consisted of studies that implemented neuropsychological tasks to probe decision-making, the most common of which is the Iowa Gambling Task (Bechara et al., 1994). In recent years, development and implementation of novel value-based decision-making tasks, including feedback learning and food choice tasks, and methodology, including neuroimaging and computational modeling, has grown, necessitating an update to refine collective knowledge of this executive function in individuals with AN. Second, separation of samples consisting of acute, weight-recovered, and remitted patients may inform relationships between illness state and differences in decision-making. Third, developmental stage has yet to be considered in a systematic review of decision-making, and may be a crucial factor to consider, as AN typically onsets during adolescence. Fourth, AN is heterogeneous, with restricting (AN-R) and binge/purge (AN-BP) subtypes possibly displaying disparate temperamental traits that may influence behavior and choices. We therefore aim to describe decision-making in AN utilizing a descriptive framework that emphasizes the characteristics described above spanning gambling, intertemporal choice, food choice, risky decision-making, and reversal learning tasks, among others. Of note, although we recognize that choice behavior utilizes and may overlap with other executive functions (e.g., cognitive flexibility, set-shifting, working memory; Wildes et al., 2014) in order to maintain focus on value-based decision-making, we do not include tasks that primarily focus on these other executive functions.

## 2. Methods

This review adhered to the guidelines outlined in the Preferred Reporting Items for Systematic Reviews and Meta-Analysis (PRISMA; Page et al., 2021). These guidelines include a checklist of recommended items to be reported (PRISMA check list) and a four-step flow diagram (PRISMA flow diagram). This systematic review was not preregistered.

## 3. Eligibility criteria

Inclusion criteria for the review were: (a) sample of participants with current/acute, weight-restored, or recovered AN, (b) cross-sectional or longitudinal study design, (c) empirical assessment of decision-making domains, operationalized using paradigms characterized by value-based choice, (d) inclusion of quantitative data, (e) inclusion of human subjects.

Exclusion criteria were: (a) use of only self-report questionnaires for diagnosis of AN or inclusion of subjects without clinical AN, as determined by DSM-IV or DSM-5 criteria, (b) papers published in a non-English language, (c) lack of quantitative data (e.g., qualitative analyses), (d) papers published before 2000, (e) systematic reviews or meta-analyses, (f) clinical trials, (g) unpublished theses, (h) conference abstracts, (i) preprints.

## 4. Information sources

A systematic literature review of PubMed and PsycINFO was conducted separately to obtain searches up to late May 2024. References of relevant manuscripts were reviewed for eligible papers that were not obtained through the databases. All results were collated, and duplicates were removed.

## 5. Search strategy

Search terms included “decision” and “decision-making”, crossed with “anorexia nervosa” (i.e., [[decision] OR [decision-making]] AND [anorexia nervosa]). Searches were also conducted using terms specific to neuropsychological tasks probing decision making, including “Iowa Gambling Task” and “Delay Discounting”, crossed with “anorexia nervosa” (i.e., [[Iowa Gambling Task] OR [Delay Discounting]] AND [anorexia nervosa]).

### 5.1. Study selection

PRISMA guidelines were followed for study selection. This included initial screening of abstracts and paper titles by two authors (first by CB and then by AN) using the inclusion/exclusion criteria, followed by full-text reviews for retained manuscripts. Inter-rater reliability was good (*κ* =.82). Disagreements were resolved through consensus between all authors.

#### 5.1.1. Quality assessment and risk of bias

Included studies were reviewed for quality and risk of bias using guidelines from the *STrengthening the Reporting of OBservational studies in Epidemiology* (STROBE) statement (Vandenbroucke et al., 2007). The STROBE checklist consists of 18–22 items, depending on the type of study being examined, and is designed to assess for transparent reporting in each section of a manuscript, including: title, abstract, introduction, methods, results, and discussion. Each study included in this review was assessed according to this checklist and given a score, calculated as the percentage of total STROBE criteria met. Each item was coded as a 1 if the study met the criterion indicated in the checklist, a 0 if it did not, or NA if the item was not relevant to the study being assessed. Overall quality of each study was given an A-C rating, defined as the following: A) 80 % or more of criteria fulfilled, B) 50–80 % of criteria fulfilled, C) less than 50 % criteria fulfilled. This method has been applied in several prior reviews (Howard et al., 2020; Olmos et al., 2008). If a study did not meet at least 50 % of the STROBE criteria, it was excluded.

## 6. Data extraction

The following data were extracted from each study: author names, publication date, population studied, number of participants (*n*) in each group, percentage of female participants, participants’ stage of illness, average age of each group, developmental stage of participants, average duration of illness (for clinical groups only), average BMI of each group, and decision-making task utilized. We coded developmental stage as “adult” if participant age was specified as >18 years, “adolescent” if participant age was specified as <18 years, and “mixed” if the age range reported consisted of ages <18 and >18 years. If the range of ages was not clearly defined, it was coded as “Not defined”. Illness stage was coded as it was reported in each study. For studies that report samples in weight-restored and remitted/recovered stages, we describe the criteria each study reported to define achievement of this stage in the Results section. This was conducted by the first author and reviewed by the remaining authors.

## 7. Summary measure

The principal summary measure was performance on decision-making tasks. Performance metrics varied by task but are described as needed below in task descriptions.

## 8. Results

**Fig. 1.**
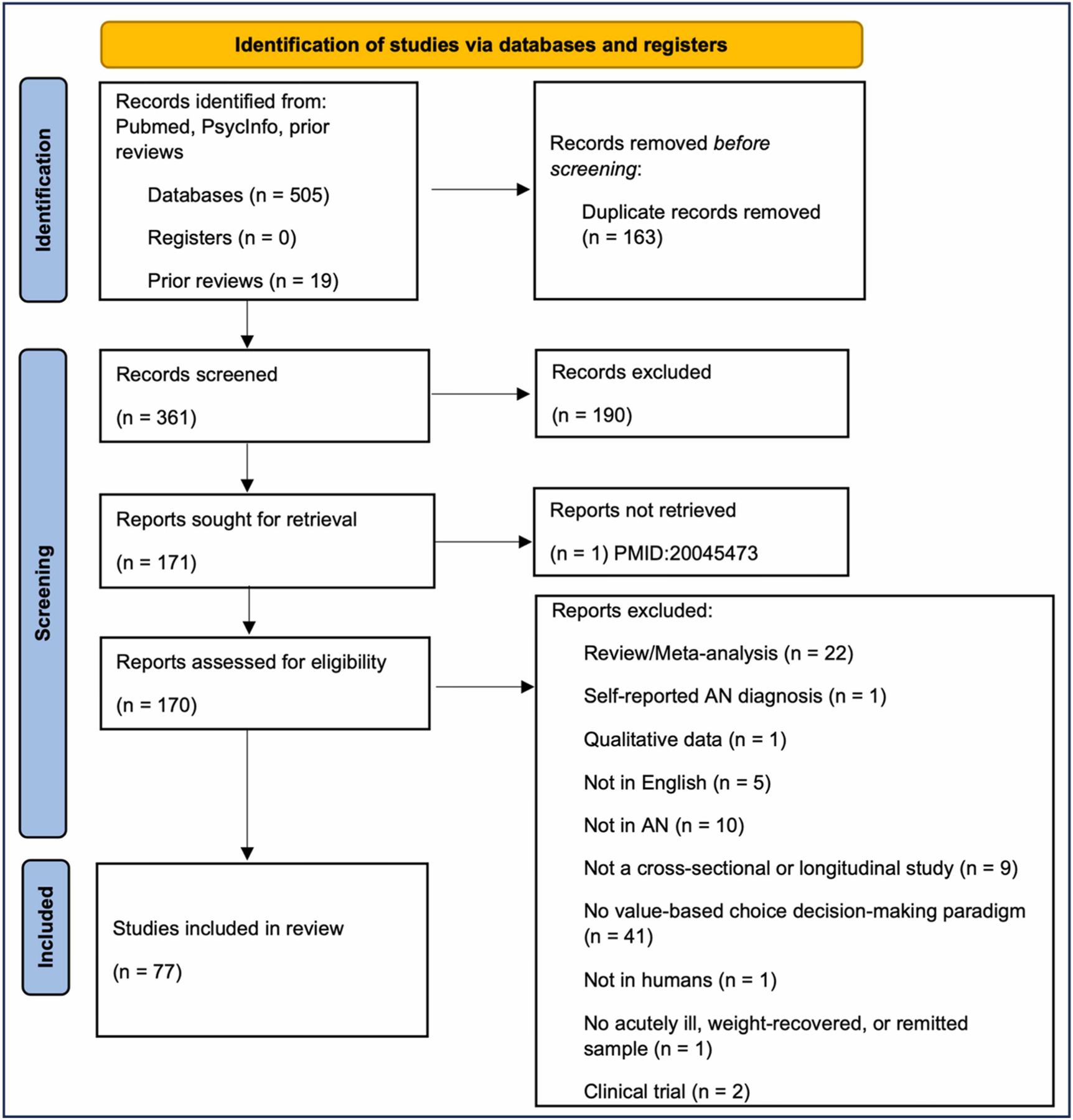
PRISMA figure. AN – anorexia nervosa.

Seventy-seven articles met criteria for the current review. Details about the studies, including sample characteristics and tasks are listed in Tables 1 – 6. A summary of the article selection procedure is described in Fig. 1. We identified 524 articles using the search terms described in the methods section. Nineteen were identified from prior reviews. One hundred sixty-three were duplicates and were removed. Of the remaining studies, 190 were screened out for irrelevance based on their titles. Finally, 93 were removed from the final sample, as they met at least one exclusion criterion, leaving 77 total studies. All 77 studies met at least 50 % of the STROBE criteria and were consequently retained. Collectively, we found that many of the studies included relatively small samples sizes, did not justify their sample sizes (e.g., reporting on power analyses), included only female participants, and used heterogeneous outcome measures even when using the same tasks. We describe the results below by grouping studies based on type of task administered and highlighting findings related to illness stage, AN subtype, and age group, if adequate information is available, within each section. Definitions of weight-restoration and remitted status differed amongst the studies and will be described in the relevant sections.

### 8.1. Gambling tasks

Gambling tasks, particularly the Iowa Gambling Task (IGT; Bechara et al., 1994), are some of the most commonly used measures of decision-making across neuropsychiatric conditions. The IGT involves selecting cards from one of four decks, all of which are associated with differing reward schedules. Specifically, two of the decks are advantageous (i.e., associated with low rewards but lower future losses, leading to a positive net score) and two are disadvantageous (i.e., associated with high rewards but higher future losses, leading to a negative net score). The IGT offers a way to simulate real-life decision-making involving uncertainty, long-terms goals, and goal-directed action. Participants must consider the balance of rewards and punishments linked to each deck and incorporate feedback to maximize gains and minimize losses, allowing for evaluation of riskiness, as well as reward and punishment sensitivity, in their behavior. Studies utilizing this task are listed in Table 1.

#### 8.1.1. Acutely ill

Studies examining performance during the IGT in acutely ill samples show poorer performance relative to comparator groups, particularly in adults (Adoue et al., 2015; Brogan et al., 2010; Danner et al., 2016; Di Lodovico et al., 2022; Elzakkers et al., 2016; Fagundo et al., 2012; Galimberti et al., 2013; Giannunzio et al., 2018; Miranda-Olivos et al., 2021; Paslakis et al., 2019; Steward et al., 2016, 2019; Verharen et al., 2019; Yokokura et al., 2019), mixed samples of adolescents and adults (Perpiñá et al., 2017; Tenconi et al., 2016, 2021), and undefined age ranges (Abbate-Daga et al., 2011; Aloi et al., 2015; Bodell et al., 2014; Cavedini et al., 2006; Chan et al., 2014; Danner et al., 2012; Garrido and Subirá, 2013; Tchanturia et al., 2007, 2012; Testa et al., 2021), with few exceptions (Bosanac et al., 2007; Guillaume et al., 2010; Jollant et al., 2007; Matsumoto et al., 2015, 2017). However, it should be noted that Jollant et al. (2007) compared performance in individuals with AN to other clinical groups, but did not include a control group. Although adult samples tend to demonstrate this deficit, adolescents’ decision-making may still be intact, even during an acute stage of illness.

Only two studies explicitly examined performance in adolescent groups and reported mixed findings (Fornasari et al., 2014; Giannunzio et al., 2018). Fornasari et al. reported reduced IGT net scores in adolescents with AN compared to their healthy control (HC) counterparts, but no differences in latent decision-making processes estimated with the expectancy-valence model (EV; Busemeyer and Stout, 2002). Giannunzio et al., on the other hand, reported the opposite, with no group differences in net scores, but greater attention to losses as estimated with the same EV model. Giannunzio et al. also noted that HC adults exhibited a trend towards significantly better net scores compared to HC adolescents, yet no such trend was seen in individuals with AN, suggesting the presence of altered developmental processes that may contribute to alterations in performance seen in adults with AN.

Seven studies directly examined differences in gambling task performance between subtypes (i.e., AN-R vs AN-BP), with mixed results (Cavedini et al., 2004, 2006; Danner et al., 2016; Garrido and Subirá, 2013; Miranda-Olivos et al., 2021; Verharen et al., 2019; Yokokura et al., 2019), including one study that implemented a separate gambling task consisting of risky and safe options (Neveu et al., 2016). Two studies reported attenuated performance in individuals with AN-R relative to individuals with binge/purge diagnoses, including greater preference for disadvantageous decks (Cavedini et al., 2004) and significantly lower net scores with no improvement in performance over the course of the task (Garrido and Subirá, 2013). Conversely, two studies reported that individuals with AN-BP demonstrated attenuated performance and failed to choose advantageous decks over time (Danner et al., 2016; Miranda-Olivos et al., 2021); however, one included a negative affect mood induction prior to the task, so results must be interpreted within the context of mood (Danner et al., 2016). The remaining three studies reported equal deficiencies in task performance between individuals with AN-R and AN-BP, with no group differences in: net scores (Cavedini et al., 2006; Verharen et al., 2019; Yokokura et al., 2019), attenuated loss-aversion (Verharen et al., 2019), and increased loss-aversion in response to food cues relative to neutral cues (Neveu et al., 2016). Importantly, the lack of group distinction in sensitivity to losses may point to a general deficit tied to AN pathology more broadly during acute illness. Although, Verharen et al.’s results are in conflict with results from Giannunzio et al., who reported higher sensitivity to loss in adolescents, and Chan et al. (2014), who reported no differences in loss-aversion or feedback sensitivity in an undefined age range. That said, these studies applied disparate models to estimate parameters of interest (e.g., loss-aversion), making comparison between studies challenging.

#### 8.1.2. Weight-restored

Only three of the listed studies included comparisons of individuals weight-restored from AN (W-AN; Bodell et al., 2014; Bosanac et al., 2007; Giannunzio et al., 2018). Bosanac et al. defined weight-restoration as achieving a BMI of ≥18.5 for at least 3 months and found no significant difference in performance between individuals with acute AN, individuals with W-AN, individuals with bulimia nervosa, and HC. The sample sizes for each group were quite small (*n*s = 12–16), so it is possible that the study was underpowered to detect differences in task performance. Giannunzio et al. defined W-AN as patients with lifetime AN who were in treatment but within a normal weight range (BMI ≥18.5). The authors reported similar deficits in individuals with W-AN as compared to individuals with acute AN in both adolescent and adult groups. Finally, Bodell et al. was the only study that included neuroimaging and the only study that had a longitudinal design, with weight restoration being defined as achievement of BMI ≥18.5 for at least one week. Consistent with Giannunzio et al., there was no significant difference in IGT performance at weight-restoration compared to performance during acute illness. And while low BMI and reduced left medial orbitofrontal cortex (mOFC) volume were associated with deficits in decision-making during acute illness, these relationships were no longer significant at weight-restoration. Altogether these findings tentatively show no substantial difference in performance at weight restoration, even compared to acute illness, however differences in definitions of weight restoration, differences in study design (e.g., cross-sectional versus longitudinal), and the meager number of studies suggest that caution must be taken when making conclusions about this illness stage.

#### 8.1.3. Remitted/recovered

Five studies were conducted in individuals recovered from AN (R-AN; Danner et al., 2012; Di Lodovico et al., 2022; Lindner et al., 2012; Steward et al., 2016; Tchanturia et al., 2007), with most operationalizing recovery as BMI>18.5 and regular menses for one year, as well as no considerable eating pathology cognitions. Danner et al. reported no difference between individuals with acute AN and R-AN; additionally, individuals with R-AN had worse IGT scores compared to HC. This study, however, is potentially limited by the small sample sizes of the groups (*n*s = 15–16) and may be underpowered to detect differences. These results were also inconsistent with the remaining four studies which all reported no significant difference in individuals with R-AN compared to HC. In fact, Steward et al. reported that patients who met criteria for partial remission or no remission at a 1-year follow-up timepoint maintained poor performance compared to the HC group. Individuals fully recovered from AN not only improved performance from acute illness to recovery, but performed similarly to the HC group, indicating that remediation of task performance may primarily occur with full remitted status. There is evidence of this change physiologically as well, with Tchanturia et al. demonstrating that anticipatory skin conductance response before selection of a card and after losses was attenuated in an acutely ill group, but not in the R-AN group. Finally, while Di Ludovico et al. found that individuals with R-AN had net scores that did not significantly differ from the HC group, they reported reduced feedback sensitivity in both individuals acutely ill with AN and R-AN. Although more research is needed, this study potentially indicates that, despite improved net scores on the gambling task, latent decision-making processes may still be altered even after recovery.

#### 8.1.4. Summary of gambling task studies

There appears to be substantive evidence of poorer IGT performance in individuals acutely ill with AN compared to HC groups, however evidence of this difference in adolescents and between AN subtypes is mixed and in need of more research. These mixed findings are made more difficult to disentangle given the heterogeneity in task metrics and models used to quantify behavior. Preliminarily, it appears that task performance is not remediated at weight-restoration, however the small number of studies examining this illness stage prevents conclusions from being made. Performance at recovery, however, appears to improve with regards to observed choice behavior. Whether that improvement extends to latent decision-making processes is still in question, given that one study reported reduced feedback sensitivity in their remitted sample. More research is needed to confirm and replicate this finding.

### 8.2. Intertemporal choice task

Intertemporal choice tasks, or delay discounting, derive from behavioral economics and test an individual’s capacity for delayed gratification. During these tasks, participants are offered the option to choose between receiving a smaller immediate reward or a larger reward that will be delivered after a delay. By adjusting the amount of reward and the duration of the delay, it is possible examine how individuals differ in their inclination to opt for smaller immediate rewards versus larger delayed rewards, which can elucidate differences in temporal decision-making and goal-directed behavior. Studies utilizing this task are listed in Table 2.

#### 8.2.1. Acutely ill

Ten studies examined delay discounting and intertemporal choice in individuals acutely ill with AN (Bartholdy et al., 2017; Bernardoni et al., 2020, 2023; Decker et al., 2015; King et al., 2016; Ritschel et al., 2015; Steinglass et al., 2012, 2017; Stern et al., 2024; Steward et al., 2017). While none of the listed studies were conducted explicitly in adolescents with AN, several recruited a wide age range of participants that included adolescence. However, due to the mixture of adolescents and adults, conclusions about developmental features of intertemporal choice tasks are limited. Of the ten studies, five found no group differences in temporal discounting (preference for immediate over future rewards) in individuals with AN compared to HC (Bartholdy et al., 2017; Bernardoni et al., 2020, 2023; King et al., 2016; Ritschel et al., 2015). Despite the mixed age range, these studies included either relatively younger or older AN samples, with mean ages of 30.0, 16.1, 16.0, 15.3, and 15.7 years respectively. Meanwhile, five found that individuals with AN showed decreased temporal discounting (i.e., greater preference for delayed reward) compared to HC (Decker et al., 2015; Steinglass et al., 2012, 2017; Stern et al., 2024; Steward et al., 2017). The relative inconsistency in the studies described here may be attributable to the differences in ages between samples or the variation in operationalization of discounting metrics, with some studies calculating “indifference points” between smaller-sooner and larger-later rewards, and others using hyperbolic discount rate k to quantify how much participants preferred smaller-sooner versus larger-later rewards. Thus, the results may be confounded by the effects of age or illness duration and methodology. Additionally, although coded as acutely ill here, mean BMI for individuals with AN was 18.5 (*sd* = 2.2) in Stern et al., suggesting that part of their sample may be at various stages of weight-restoration.

Three studies explored potential differences in AN subtypes, with all finding that reduced temporal discounting was largely driven by individuals with AN-R (Decker et al., 2015; Steinglass et al., 2012; Steward et al., 2017). Although all studies found that individuals with AN, as a whole, displayed attenuated temporal discounting, post hoc analyses revealed that individuals with AN-BP had a mean discount factor that was not significantly different compared to HC. These studies demonstrate that individuals with AN-R may be especially characterized by extreme self-control, while individuals with AN-BP may not be in all domains.

Two studies reported differences in neural functioning associated with intertemporal choice in individuals with AN (Decker et al., 2015; King et al., 2016). Contrary to predictions of greater prefrontal cortex activation in response to delayed reward, Decker et al. reported that individuals with AN showed less activity than HC during delayed choices in the dorsal anterior cingulate cortex and striatum. They hypothesized that self-control in AN may not be driven by increased prefrontal cortex activation, but rather by differences in striatal functioning. King et al., however, found decreased activity in frontoparietal regions associated with executive functioning and control, and increased activity in the bilateral posterior thalamus and right middle occipital cortex, perhaps strengthening theories that these results reflect “neural efficiency” guided by an over-reliance on habitual responding in individuals with AN, as opposed to singular reliance on enhanced top-down cognitive control.

#### 8.2.2. Weight-restored

Of three studies investigating intertemporal choice in individuals with W-AN, all included a longitudinal aspect to their study design, with two reporting no significant improvement in task performance at weight restoration (Doose et al., 2020; Ritschel et al., 2015), and one reporting improvement (Decker et al., 2015). However, the two studies reporting no significant improvement assessed performance at partial weight-restoration, operationalized as an increase in BMI of ≥10 % and ≥12 %, respectively. Despite the lack of improvement at partial weight-restoration, Doose et al. demonstrated that default-mode network activation strengthened at the second time-point, possibly indicating that neural changes in self-referential decision-making may correspond with weight rehabilitation. Meanwhile, weight-restoration in Decker et al. was operationalized as an increase in BMI from admission to ≥19.5 kg/m^2^. Individuals with W-AN performed the task similarly to HC, however they showed increased neural activity in the striatum, dorsal anterior cingulate cortex, and right dlPFC in response to delayed versus sooner choices, a finding opposite to HC.

#### 8.2.3. Remitted/recovered

Three studies included investigations of individuals with R-AN using intertemporal choice tasks (Bernardoni et al., 2020; King et al., 2020; Wierenga et al., 2015), with most defining recovery as a BMI>18.5 for adults or BMI>10th percentile for participants under 18 years old, regular menses, and no disordered eating behavior for at least 6 months; Wierenga et al. was the same, except that weight was defined as >85 % of average body weight for at least one year. All studies indicated that individuals with R-AN did not differ from HC in task performance, with one also showing no differences in neural activation during the task compared to HC (King et al., 2020). Despite lack of behavioral differences, Bernardoni et al. found that individuals with R-AN displayed risk-aversive behavior, characterized by higher preferences for smaller but more certain rewards, and Wierenga et al. reported lack of differentiation between hunger and satiety in task-elicited decision making neural response, as well as state-independent elevation of dlPFC activity. Yet again, while task performance itself was not necessarily impaired in recovered individuals, elevations in cognitive control circuitry, regardless of metabolic state, reflect differences in biological processes that may be artifacts from illness or reflect premorbid neural factors.

#### 8.2.4. Intertemporal choice task summary

Evidence of alterations in delay discounting during acute illness was inconsistent, with half of the studies reporting no difference compared to controls and half reporting increased delay discounting. Beyond the heterogeneity in performance metrics and age groups, one potential influence could lie in the lack of distinction between AN subtypes. Studies that investigated differences in subtypes showed initial evidence that reduced delay discounting may be primarily driven by AN-R and may not be as prominent in AN-BP, meaning that studies collapsing the subtypes may be masking effects that are unique to AN-R. With regards to neural findings, delay discounting was not shown to be associated with greater dlPFC activation, contrary to hypotheses about superior top-down control during acute illness. This potentially raises questions about how cognitive control and effortful control are represented in the brain during acute illness, specifically whether these results indicate some form of neural efficiency, or even whether delaying rewards requires effortful control in this population. Notably, at weight-restoration one study did find increased activation in response to delayed choices in dlPFC and improved task performance, while another reported increased dlPFC activity that was not sensitive to metabolic state at recovery. The inconsistency in PFC activity across illness stages may consequently reflect the changes in cognitive control and effortful control inherent with improvement of symptoms, possibly consistent with the idea that making the choice to delay reward is easier at acute stages but becomes more difficult as recovery progresses. More research is needed to examine this possibility and replicate neural findings.

### 8.3. Food choice task

While much research has focused on monetary reward, relatively few studies have examined decision-making in other domains. In the context of AN, the most disorder-relevant outcome in day-to-day decision-making is associated with food. To better understand why individuals with AN make maladaptive food choices, a modified food choice task (Hare et al., 2009), involving the presentation of “high-fat” and “low--fat” food images, was developed (Steinglass et al., 2015). Participants rate each item on perceived healthiness and tastiness. Following the ratings, participants are asked to choose between two food items across multiple trials, one of which is a trial-unique food item and the other always being a neutral “reference” food item. Thus, the task elicits participants’ perceptions of healthiness and tastiness of the stimuli, as well as individual proportions of high-fat to low-fat choices. Studies utilizing this task are listed in Table 3.

#### 8.3.1. Acutely ill

Eight studies were conducted in individuals acutely ill with AN, the majority of which came from the same research group and included mixed samples of adults and adolescents (Cowdrey et al., 2013; Dalton et al., 2020; Foerde et al., 2015, 2022; Foerde, Walsh, et al., 2021; Steinglass et al., 2015; Uniacke et al., 2020; Xue et al., 2022). One study reported individuals with AN had slower reaction times when selecting high-calorie foods, which was interpreted as reduced ‘implicit wanting’ (Cowdrey et al., 2013). Others showed that, overall, individuals with AN were less likely to choose high-fat foods compared to HC, with taste ratings declining with increased length of illness (Steinglass et al., 2015), were influenced by both taste and health ratings when making food choices (Foerde et al., 2022; Steinglass et al., 2015), and showed high-fat choice behavior during the task that correlated with caloric intake at lunch the day after (Foerde et al., 2015, 2022). The authors posited that alterations in food value computations may explain why palatable food is no longer hedonically rewarding for individuals with AN, with a behavioral consequence of aberrant valuation manifesting as repeated choice of low-fat food. Additionally, both Steinglass et al. and Uniacke et al. investigated the food choice paradigm in individuals with AN-R compared to AN-BP, with both studies finding no difference between AN subtypes. Thus, maladaptive food choice may be a general phenotype of AN and may not be modulated by the presence of binge/purge symptomatology.

Six of these studies examined neural mechanisms contributing to food choice (Dalton et al., 2020; Foerde et al., 2015, 2022; Foerde, Walsh, et al., 2021; Muratore et al., 2021; Xue et al., 2022). Food choice was found to be associated with greater dorsal striatum activity compared to HCs (Foerde et al., 2015, 2022; Foerde, Walsh, et al., 2021), with greater connectivity to dorsolateral prefrontal cortex (dlPFC) when faced with low-fat relative to high-fat food stimuli, but not necessarily greater dlPFC activation during food choice blocks. Additionally, Xue et al. reported that OFC activity related to health ratings was more strongly associated with how much participants preferred high-fat or low-fat items versus the reference (neutrally-rated) items compared to the HC group. Overall, there is preliminary evidence that frontostriatal circuitry is particularly implicated in food-related decision-making during acute illness, consistent with models of AN pathology that stress the relevance of altered reward and habit systems in the disorder (O’Hara et al., 2015).

#### 8.3.2. Weight-restored

Only one study utilized the food choice paradigm longitudinally in individuals with W-AN, which was operationalized as BMI=19.5–20.5 (Foerde, Walsh, et al., 2021). Individuals with AN did not show changes in health or taste ratings, nor changes in the frequency with which they chose low-fat foods over high-fat, at weight-restoration. Although there was limited improvement in choice behavior, at the second timepoint caudate activation was similar to HC, demonstrating potential neural changes that might precede behavioral improvement.

#### 8.3.3. Remitted/recovered

Cowdrey et al. (2013) also included a remitted sample, defined as maintenance of BMI=18.5–25 for one year, eating pathology measured within one standard deviation of mean scores for young women, and no current DSM-IV psychiatric disorder. Although there was no significant group difference for explicit wanting (e.g., when asked to rate how much they want the food), implicit wanting of high calorie foods (e.g., faster reaction times) was greater for R-AN compared to the acutely ill group. Moreover, the acutely ill group wanted low calorie foods significantly more than R-AN, suggesting that motivational processes can be remediated.

#### 8.3.4. Food task summary

Results from studies in acutely ill samples show support for lower choice of high-fat food options compared to HC groups, which was not differentiated by AN subtype. This behavior possibly represents one area of decision-making that is generalized across different presentations of AN. Food choice was associated with higher activity in dorsal striatal regions for AN relative to HC groups, with increased connectivity to dlPFC during trials with low-fat food items, interpreted as involvement of habit processes. Notably, at weight-restoration, activity in dorsal striatal regions during food choice was no longer significantly different compared to controls, despite lack of change in choice behavior, providing initial evidence that change in frontostriatal functioning may be an important precursor to behavioral change, or possibly that food choice behavior is extremely resistant to change despite shifts in neural functioning. More longitudinal research is needed in recovered samples to determine whether food choice can be remediated.

#### 8.3.5. Wheel of fortune task, beads task, and balloon analogue risk task

The wheel of fortune (WoF) task, Beads task, and balloon analogue task (BART) were designed to evaluate decision-making under conditions of uncertainty and risk (Ernst et al., 2004; Huq et al., 1988; Lejuez et al., 2002). In the WoF, participants are presented with a choice between two wheels, each with differing probabilities of receiving monetary reward and with the goal of maximizing reward over the course of the task. Three conditions are used to elicit risk-taking and risk-aversive behavior, including selection between the following sets of wheels: (1) a 50 % chance of loss vs. a 50 % chance of gain, (2) a 30 % chance of gain vs. a 70 % chance of loss, and (3) a 10 % chance of gain vs. a 90 % chance of loss. Choices are riskier or safer, with differing amounts of reward possible for each option.

The Beads task was specifically designed to operationalize an information processing bias characterized by hasty decision-making or a “jumping to conclusions” bias associated with delusions. Beads of two different colors are drawn from two jars, each with a specific ratio of one color bead to another (e.g., 85:15 ratio in jar 1, 15:85 ratio in jar 2). Participants decide when they have seen enough draws to accurately determine which jar the beads are being drawn from. A common variation to this task includes different sets of ratios that manipulate the level of difficulty, and consequently uncertainty, in the task.

Similarly, the BART operationalizes decision-making under uncertainty by having participants inflate a balloon (associated with incremental awards of money) until they decide to stop and collect the winnings. If the balloon pops, they do not receive money.

Studies utilizing these tasks are listed in Table 4.

#### 8.3.6. Acutely ill

Two studies from the same research group utilized the WoF task in individuals acutely ill with AN, with both observing that reaction times were significantly slower under conditions of maximum uncertainty (50/50 wheel) compared to HC, suggesting greater caution during conditions of uncertainty (Piccolo et al., 2019a; Piccolo et al., 2019b). However, these studies are limited by very small AN sample sizes (*n*s = 12 – 17).

Three studies applied the Beads task, with two being conducted in adult samples and one being conducted in a mixed adolescent and adult sample (McKenna et al., 2014; Sternheim et al., 2011; Wittorf et al., 2012). All three reported no significant difference in the number of beads requested in individuals with AN compared to the healthy comparison groups, suggesting no overt alterations in hasty decision-making.

With regards to the BART (Adoue et al., 2015; Jenkinson et al., 2023; Neveu et al., 2016), both Adoue et al. and Jenkinson et al. found that adults with AN made significantly fewer risky choices than HC. However, Jenkinson et al. modified the BART to include conditions with a female body avatar that either increased or decreased in size and found that, although individuals with AN made fewer risky decisions and behaved as if the probability of losing was high compared to HC, they paradoxically made riskier decisions for the decreasing body size condition compared to a decreasing balloon condition, suggesting that they are willing to take greater risk when outcomes may result in reduced body size. To compare subtypes, Neveu et al. introduced preconditioning situations involving neutral, food-related, and stressful imagery that were presented before each trial of the BART in individuals with AN-R, individuals with AN bingeing type (AN-B), individuals with bulimia nervosa, and HC. Individuals with AN-R and bulimia nervosa displayed similar conservative behavior during the food compared to neutral condition, while individuals with AN-B were behaviorally similar to HC.

#### 8.3.7. Weight-restored

Piccolo et al. (2019b) conducted a longitudinal study where the WoF was administered during acute illness and again at weight-restoration, defined as reaching a BMI of ≥17.5. Following weight restoration, individuals with W-AN displayed no significant difference in reaction time during conditions of maximum uncertainty compared to HC, providing preliminary evidence that sensitivity to uncertainty may improve with weight restoration; however, more work is needed to replicate these findings.

#### 8.3.8. Remitted/recovered

Jenkinson et al. (2023) included an R-AN group, in addition to the acutely ill group described above, where remitted status was defined as BMI>16.5, regular menses, and no disordered eating behaviors for at least 6 months. Although the sample mean BMI was >18.5, the presence of lower weight participants should be noted. Like the acutely ill group, individuals with R-AN displayed attenuated risk-taking behavior during the BART, particularly for the increasing body size compared to the balloon condition, but greater risk-taking behavior for the decreasing body size condition. Individuals with R-AN displayed higher probability of loss beliefs, as did the acutely ill group, in addition to reduced risk aversion for all conditions compared to HC, but no differences in loss aversion.

#### 8.3.9. Wheel of fortune task, beads task, and balloon analogue risk task summary

Overall, individuals acutely ill with AN show increased sensitivity to uncertainty, which did not improve at weight-restoration in one study, and reduced risk-taking behavior, but no alterations in hasty decision-making. One potentially important and surprising finding indicated that, while individuals with AN tended to make less risky decisions overall, they displayed greater risk-taking behavior for disorder-consistent contexts (i.e., decreasing body size) during both acute and recovered stages of illness. While this is in need of replication, the results subvert the conceptualization of individuals with AN as rigidly harm avoidant and risk averse across all contexts and support the notion that risk-taking behavior may be domain-specific and resistant to change.

### 8.4. Probabilistic reversal learning tasks

Probabilistic reversal learning tasks are typically characterized by choices between a set of stimuli, each with differing probabilities of a rewarding or aversive outcome (e.g., a choice between a circle and square, associated with 80/20 probabilities of reward) that reverses contingencies throughout the task. These tasks typically probe for cognitive flexibility in choice behavior, particularly ability to shift behavior in response to changes in environmental contingencies. Studies utilizing these tasks are listed in Table 5.

#### 8.4.1. Acutely ill

Adoue et al. (2015) reported no group differences during a probabilistic reversal learning task in a sample of adults with AN, consistent with studies in mixed age ranges (Bernardoni et al., 2017; Geisler et al., 2017). Despite this, two studies reported greater neural activity in response to negative feedback in the AN group relative to HCs, one in the dorsal anterior cingulate cortex (dACC; Geisler et al., 2017) and one in the medial prefrontal cortex (mPFC; Bernardoni et al., 2017). Further, individuals with AN showed a tendency to shift choices after receiving negative feedback compared to HC, which was associated with greater connectivity between dACC and amygdala (Geisler et al., 2017), and showed higher learning rates following punishment (Bernardoni et al., 2017). These studies may therefore be indicative of latent cognitive and neural hypersensitivities to negative outcomes in acute illness, despite no apparent differences in raw behavior or task performance.

Only one study investigated altered reversal learning in adolescents with AN, finding poorer performance compared to HC (Sarrar et al., 2011). This study was longitudinal and included examination at a second time point once patients reached a BMI greater than or equal to the third percentile, however mean BMI was still <18.5 (*M* = 17.4; *SD* = 0.7). Although performance improved and total score was not significantly different compared to HC at the second time point, adolescents with AN more often found but ignored correct choices.

#### 8.4.2. Remitted/recovered

Two studies utilizing reversal learning in remitted samples reported lower contingency reversals and correct trials in individuals with R-AN compared to HC (Bernardoni et al., 2021; Ritschel et al., 2017). In both, recovery was operationalized as maintaining a BMI>18.5 for adults (or BMI>10th percentile for participants under 18 years old) for at least 6 months (Ritschel et al., 2017) or 9 months (Bernardoni et al., 2021), regular menses, and no pathological eating behaviors. Consistent with studies in acute illness, individuals with R-AN displayed higher lose-shift behavior compared to the HC group, associated with greater activation in regions linked to the frontoparietal network, including bilateral angular gyrus, left inferior frontal junction, and right OFC (Ritschel et al., 2017). Bernardoni et al. (2021) found a larger difference between negative and positive learning rates in individuals with R-AN compared to HC and greater mPFC activation following negative outcomes, which was also present in individuals with acute AN. Together, these studies indicate that sensitivity to punishment may be a stable trait in individuals with AN or difficult to extinguish.

#### 8.4.3. Probabilistic reversal learning task summary

Despite limited evidence of alterations in observed task performance during acute illness, individuals with AN showed differences in latent decision-making or subprocesses of decision-making, particularly processes associated with negative feedback. Specifically, these initial results show faster learning rates following punishment in individuals acutely ill with AN, as well as greater lose-shift behavior compared to controls at both acutely ill and recovered stages. This sensitivity to negative outcomes extends to neural activation, with studies finding hyperresponsivity in dACC and mPFC in response to negative feedback during acute illness, and increased activation in frontoparietal regions associated with lose-shift behavior in recovered stages. While initial evidence supports the notion that processing of negative feedback may be a particularly critical mechanism in choice behavior and decision-making for individuals with or recovered from AN, conclusions would be strengthened by more studies investigating latent decision-making processes involved in reversal learning.

### 8.5. Other tasks

Here, we describe studies that utilized more novel tasks that have yet to be replicated in individuals with AN. Specifically, we include a probabilistic associative learning task, monetary guessing task, acquired equivalence task, two-step sequential decision-making task, slips of action task, volatility task, ultimatum game, and a self-referential encoding task. Studies utilizing these tasks are listed in Table 6.

#### 8.5.1. Acutely ill

Five studies included samples of individuals with acute AN (Bischoff-Grethe et al., 2013; Godier et al., 2016; Gu et al., 2024; Isobe et al., 2018; Wierenga et al., 2021). Wierenga et al. (2021) implemented a probabilistic associative learning task under conditions of reward and punishment in individuals with acute and partially weight-restored AN-R. During the task, participants were prompted to indicate whether a presented cue belonged to category “A” or “B”, with two of four stimuli being optimal and the remaining two being non-optimal. During the reward condition, correct responses resulted in monetary gain, while incorrect responses resulted in no change. During the punishment condition, correct responses resulted in no change, while incorrect responses resulted in monetary loss. The authors demonstrated that individuals with AN-R, compared to HC, displayed lower learning rates for both positive and negative prediction errors (i.e., the difference between the received outcome and the expected outcome) and showed deficits in the utilization of cue valuation to make choices, resulting in higher stochasticity in their decision-making. Therefore, not only were individuals with AN-R characterized by difficulty updating expectations following errors, but they also displayed weaker deterministic choice behavior, perhaps as a result of reductions in decisiveness, lack of trust in changing valuation of choices, or impairments in incorporation of feedback into decisions.

Godier et al. (2016) applied a slips of action paradigm designed to investigate goal-directed and habit behavior. The task involves three stages: instrumental discrimination training, outcome devaluation test, and a slips of action test (with stimuli from the discrimination training) versus a baseline test (where stimuli are devalued instead of outcomes). Habit behavior was operationalized as perseverative actions towards stimuli with the devalued outcomes during the slips of action test. Individuals with AN did not display deficits in goal-directed and habit control compared to HC and were able to withhold inappropriate responses following outcome devaluation.

Isobe et al. (2018) investigated social decision-making using an ultimatum game where offers of money are made and the receiver must decide whether they will accept or reject the offer. Adults acutely ill with AN offered more money to the receiver, but also expected more money to be offered when they were the receiver, suggesting greater concern about the perceived fairness of the interaction.

Gu et al. (2024) utilized a self-referential encoding task, described as a neurocognitive task designed to elicit implicit and explicit self-referential processing. Participants were presented with positive and negative descriptive terms and were tasked with deciding whether the presented word described themselves. Following the task, they completed a recall and recognition of the positive and negative words presented. Compared to HC, individuals with AN showed higher negative processing bias (i.e., higher endorsement of negative words compared to positive), higher propensity to endorse negative words as self-relevant, greater overall negative self-evaluation, and slower latent information accumulation to reject a negative word as not self-relevant, demonstrating alterations in information processing that may result in higher negative self-evaluation bias.

One study included neuroimaging and was conducted in adolescents and young adults (Bischoff-Grethe et al., 2013). Bischoff-Grethe et al. applied a monetary guessing game during fMRI, where participants were prompted to choose whether a hidden number would be less than or greater than five. Correct guesses won $2.00, incorrect guesses lost $1.00, and no choice lost $0.50. While HC exhibited responsivity in posterior putamen and motor cingulate for wins, individuals with AN failed to show valence-related differences in these regions, but did show increased activation in the posterior caudate in response to losses, consistent with other decision-making studies (e.g., reversal learning) reporting sensitivity to punishment.

#### 8.5.2. Weight-restored

Only two studies included individuals with W-AN and both were longitudinal, consisting of data collection at hospital admittance and then later at weight restoration (Foerde, Daw, et al., 2021; Foerde and Steinglass, 2017). Both defined weight-restored as achieving 90 % ideal body weight, corresponding to a BMI of approximately 19.5–20.0. Foerde & Steinglass employed an acquired equivalence task to examine feedback learning in individuals with AN compared to HC. The acquired equivalence task consisted of training and testing phases. In the training phase, participants learned to associate a set of faces with different images of landscapes. During the testing phase, they were presented with both trained associations and novel, untrained associations. Individuals with AN exhibited difficulties with feedback learning in the training phase and lower accuracy in the testing phase compared to HC, however they did not show deficits in generalization of learning to novel, untrained associations. Again, there was no improvement at weight-restoration for individuals with AN, demonstrating that deficits in feedback learning are possibly not remediated by weight restoration. The second study included a two-step sequential decision-making task, typically used to ascertain whether participants are utilizing primarily goal-directed or habit learning strategies, which map onto a class of reinforcement learning known as model-based and model-free control (Daw et al., 2011). Foerde et al. (2021) conducted two versions of this task, one utilizing monetary reward and the other using food reward. For both conditions, individuals with AN displayed reduced model-based control, which can be interpreted as attenuated utilization of goal-directed learning; this was particularly evident for individuals with AN-BP. And although this deficit improved at weight-restoration, it remained attenuated in comparison to HC.

#### 8.5.3. Remitted/recovered

Three studies included individuals with R-AN (Godier et al., 2016; Pike et al., 2023; Wagner et al., 2007), the first of which also recruited ill samples (described above). Godier et al. and Pike et al. defined recovery as maintenance of BMI>18.5 for a year, as well as low eating pathology scores; Godier et al. also specified the presence of no other current psychiatric diagnoses. Wagner et al. specified at least 85 % of average body weight, regular menses, and no eating disordered behaviors for one year. Mirroring the ill group, Godier et al. reported that individuals with R-AN did not display deficits in goal-directed or habitual control during a slips of action paradigm. Pike et al., on the other hand, reported differences in learning during a volatility task, where the volatility of outcomes was manipulated so that stimulus-outcome pairings became more dynamic and blocks were either volatile or stable. Specifically, they observed that individuals with R-AN were characterized by increased learning rate adjustment to both wins and losses in volatile environments compared to controls, indicating greater adaptability to volatility and countering hypotheses about cognitive inflexibility as a trait feature of AN. Using neuroimaging in a monetary guessing task, Wagner et al. noted increased left caudate activity in individuals with R-AN, with percentage of signal change being positively associated with trait anxiety. And while wins and losses were distinguishable in the left anterior ventral striatum for HC, individuals with R-AN did not show this distinction.

#### 8.5.4. Other tasks summary

Caution must be taken when interpreting findings relevant to this section, as many of these studies have yet to be replicated. Nevertheless, studies in acutely ill individuals show preliminary evidence of alterations in processing relevant to self- and social choices, slower learning rates that are not modulated by valence and more stochastic decision-making. In both adolescents with AN and individuals with R-AN there was evidence of increased activity in dorsal striatal regions (i.e., caudate), with adolescents specifically showing this activity in response to losses. These findings are consistent with other studies included in this review that also show sensitivity to negative feedback in individuals with AN, as well as the relative importance of dorsal striatal activity during choice behavior. Studies in weight-restored samples showed lack of improvement for both feedback and goal-directed learning. However, evidence of deficient goal-directed learning or inability to modulate learning in response to volatility was not observed at recovery.

## 9. Discussion

In this systematic review, we examined the literature on value-based decision-making in individuals with AN, with the goal of highlighting differences across illness state, developmental stage, and AN subtype in studies using disparate decision-making tasks to probe choice behavior under relevant domains (e.g., risk, reward, punishment, uncertainty). Overall, we found that the majority of studies (~44 %) utilized the IGT, however, in recent years, development and employment of more novel paradigms (e.g., food choice task) have begun to emerge, allowing for more nuanced understanding of cognitive processes that shape or are shaped by eating disorder pathology. About 15 % of the studies consisted of an intertemporal choice task, ~10 % included a food choice task, ~10 % included an uncertainty/risk task (i.e., BART, WoF, Beads), and ~6 % consisted of reversal learning tasks. Additionally, there was a heavy skew towards studies involving acutely ill, adult participants, with little differentiation between subtypes and few studies incorporating neuroimaging. We highlight these components below. Additionally, we distinguish and report on constructs that are critical to consider within the decision-making literature, including: reward/punishment, uncertainty/risk, and inflexibility/control.

### 9.1. Stage of illness

As reported above, the majority of studies were conducted with participants who were experiencing acute illness. While investigations into acute illness are critical to understand mechanisms that may initiate and maintain the disorder, exploration of decision-making in individuals with W-AN and R-AN may help differentiate between the contribution of malnutrition and/or psychological aspects of illness to decision-making differences. Studies in individuals with acute low-weight AN suggest some amount of decision-making impairment across tasks, replicating conclusions made by Guillaume et al. (2015) in their decision-making meta-analysis. Differences in decision-making during illness were evident in higher choice of disadvantageous decks in the IGT, reduced delay discounting in intertemporal choice tasks, higher choice of low-fat food items in a food choice task, slower reaction times under conditions of uncertainty in a WoF task, fewer risky choices during the BART, greater shift behavior and faster learning rates in response to loss during reversal learning, and reduced ability to update incorrect expectations and exploit cue valuation in a probabilistic associative learning task. Deficits in task behavior were accompanied by aberrant neural activity as well, with most studies finding alterations in striatal regions, particularly in response to choice. AN has been conceptualized as a disorder of extreme control, so it is perhaps surprising that greater dlPFC activation was not consistently found in individuals with AN during decision-making; yet, Decker et al. (2015) postulate that the behavioral control that is so characteristic of this disorder may be related to changes in striatal functioning, as opposed to enhanced top-down control from frontal regions.

Findings were not as clear in studies with weight-restored participants, with some studies reporting no differences compared to HC and others reporting deficits. This may, in part, be due to varying operationalizations of weight-restoration, as well as dissimilar time frames between achievement of weight-restoration and study participation. Weight-restoration is a critical stage, as it confers insights into the relative state-related importance of malnutrition and psychological characteristics of the disorder in the maintenance of aberrant decision-making during illness as well as possible state-related consequences of low weight. Therefore, standardizing how weight-restoration is defined may be particularly useful for this purpose. While there were mixed findings behaviorally, there was some preliminary evidence that weight-restoration was associated with neural changes in the striatum, particularly dorsal regions, anterior cingulate cortex, and dorsolateral prefrontal cortex, regions tied to valuation of actions and cues associated with reward (Hayden and Platt, 2010; Matsumoto et al., 2003; Samejima et al., 2005), suggesting that improvements in brain function may be a precursor to behavioral change (Decker et al., 2015; Foerde, Walsh, et al., 2021). Of note, eating disorder duration was not predictive of executive dysfunction measured with standardized neuropsychological tests in individuals with AN-R, but was predictive in individuals with AN-BP (Miranda-Olivos et al., 2021).

Behavioral deficits were largely absent in remitted samples. Indeed, the majority of studies utilizing the IGT or intertemporal choice tasks found no differences in decision-making behavior in remitted samples compared to controls, with a minority of studies reporting group differences. While behavioral choice data may indicate remediation of decision-making impairments, there is some initial evidence that latent processes are impacted, providing potential evidence of a “scar” of the illness or a trait-level feature of AN. It may be especially important to examine how neural computations and decision-making subprocesses affect choices, even when the choices made are ultimately similar to those of healthy individuals. Thus, disparate algorithmic processes may result in comparable cognitive or behavioral outcomes. Computational modeling offers one way to explore this (Haynos et al., 2022). Indeed, several studies implemented various computational models, with two finding evidence of altered feedback sensitivity and feedback learning in a remitted sample, despite no differences in their choice data when compared to HC (Bernardoni et al., 2021; Di Lodovico et al., 2022). Neuroimaging studies strengthen this finding, as Wierenga et al. (2015) reported no differences between individuals with R-AN and HC in choice behavior during an intertemporal choice task, but elevated activity in cognitive control regions that was not differentiated by states of hunger or satiety. Similarly, Wagner et al. (2008) did not find group differences in reaction time between individuals with R-AN and HC during a monetary guessing task, but showed a lack of distinction to wins and losses in the anterior ventral striatum and exaggerated caudate-dorsal striatum activation. Together, these studies illustrate how task performance may not differentiate individuals with R-AN, but altered responding to feedback both behaviorally and neurally may persist, even after recovery.

### 9.2. AN subtype

AN as a diagnostic category is associated with some amount of heterogeneity, captured by the restricting subtype, associated with an inhibitory and over-controlled temperament, and the binge/purge subtype, associated with an under-controlled temperament (Turner et al., 2014). Given the differences in phenotypic expression within AN, it is possible that decision-making processes are not identical between subtypes. However, findings were very mixed in the studies we included here. For the IGT, two studies found worse performance in individuals with AN-R, two found worse performance in individuals with AN-BP, and three found no differences between the subtypes. Meanwhile, greater delay discounting in intertemporal choice tasks was largely driven by individuals with AN-R, while deficits in goal-directed learning during a two-step sequential decision-making task was mostly driven by individuals with AN-BP. The food choice task, however, displayed no differences in choice behavior between groups, offering potentially one decision-making context that is a generalizable feature of AN.

### 9.3. Developmental stage

Unfortunately, there were a limited number of studies that investigated decision-making with a focus on adolescence and young adulthood. Because AN typically onsets in adolescence, studies that recruit this population will include participants who are closer to the onset of the disorder and with shorter durations of illness, which may provide insight into mechanisms unique to early stages of AN. Across studies that explicitly recruited adolescent samples, augmented sensitivity to punishment was commonly reported. This was noted in a study implementing the IGT (Giannunzio et al., 2018), as well as a monetary guessing task (Bischoff-Grethe et al., 2013) and a probabilistic reversal learning task (Bernardoni et al., 2021; Geisler et al., 2017), indicating that attention to and sensitivity towards punishment or losses is not task-specific and may generalize to a range of cognitive paradigms. Only Giannunzio et al. (2018) conducted the same task in adults and found similar attention to losses, yet more research is needed to understand whether these behavioral features persist into adulthood or whether developmental maturation modulates characteristics present in adolescence.

### 9.4. Reward/punishment

In concordance with Haynos et al. (2020), disentangling reward and punishment signals from behavioral and neural data proves challenging. This is in large part due to a lack of clarity on the salience of commonly utilized reward cues, such as monetary reward, in AN. The majority of the tasks included in this review used monetary reward as a means of measuring decision-making and the proceses that are central to choice behavior (e.g., learning). However, some early studies reviewed here suggest that individuals with AN show reduced neural differentiation between wins and losses, hunger and satiety, as well as a failure to elicit positive mood from immediate wins (Bischoff-Grethe et al., 2013; Piccolo et al., 2019a; Wagner et al., 2008; Wierenga et al., 2015), suggesting that known alterations in incentive salience (Sala et al., 2018) may influence value-based decision-making. In their systematic review of reward and punishment in individuals with AN, Haynos et al. established that disorder-relevant cues consistently evoked reward (e.g., restrictive eating, purging, weight loss) and punishment (e.g., normal/higher weights) responses. Within the decision-making literature thus far, food choice paradigms offer a way to investigate disorder-specific processing, and yet food cues can be wrought with reward-punishment conflict. Although not quite disorder-specific, punishment appeared to be more broadly distinguishable, with several studies reporting evidence of heightened sensitivity to punishment and altered feedback learning, particularly in adolescent and young adult samples. Only one study found that individuals with AN exhibited insensitivty to losses as compared to HC (Verharen et al., 2019). Interestingly, this study only recruited adults, whereas the studies observing increased punishment sensitivity were conducted in younger samples. Although more replication research is needed, one hypothesis for this phenomenon might be that sensitivity to loss may reflect a temperamental feature that predisposes an individual to AN, and, as the disease progresses, this sensitivity becomes avoidance of negative feedback. Supporting this, Ritschel et al., (2017) reported excessive lose-shift behavior in individuals with R-AN, although this was also evident in individuals acutely ill with AN (Geisler et al., 2017).

### 9.5. Uncertainty/risk

It is well documented that intolerance of uncertainty represents a core feature of AN that may maintain the disorder (Brown et al., 2017). High harm avoidance in this population may predispose individuals towards finding lack of certainty, or the possibility of negative or positive outcomes, to be punishing in itself (Harrison et al., 2010). Engagement in disorder-relevant behaviors, such as dieting and purging, is hypothesized to play a role as a type of coping mechanism for regulating feelings of uncertainty (Brown et al., 2017). Indeed, eating disorder behaviors can result in both disorder-consistent outcomes (e.g., weight loss) and undesired secondary (e.g., loss of concentration, dizziness) outcomes; and yet Haynos et al. (2020) noted that most of these outcomes, regardless of valence, are expected and do not confer as much uncertainty as recovery-oriented behaviors, perhaps underscoring why eating-related pathology is so entrenched and resistant to change. Findings from the current review did indicate that individuals with AN exhibited longer reaction times to less certain states, demonstrated higher aversion to risk as evidenced by lower scores on the BART, as well as worse performance during initial blocks of the IGT, when uncertainty was highest (Adoue et al., 2015; Giannunzio et al., 2018; Jenkinson et al., 2023; Piccolo et al., 2019a, 2019b). Yet, one study demonstrated that risk-taking behavior can be modulated by disorder-relevant stimuli, showing that individuals with acute and recovered AN are more prone to taking risks when the outcome involves reductions in body size. This could explain repeated restriction despite the possibly fatal outcomes and may further bolster theories suggesting that disorder-relevant reward becomes rigidly salient, even following recovery.

### 9.6. Inflexibility/control

Much research indicates that excessive control and inflexibiity are hallmarks of AN (Abbate-Daga et al., 2011; Fagundo et al., 2012; Galimberti et al., 2013), with desire for certainty and harm avoidance resulting in dependence on specific patterns of behavior or “rules” that become overly rigid and impervious to environmental changes (Merwin et al., 2011). Moreover, greater cognitive inflexibility may serve to increase inhibitory control, or suppress behavioral responses in service of particular goals, perhaps supporting the ability to forego immediate reward (e.g., palatable food) for long-term reward (i.e., weight loss). Yet, in a recent systematic review, Miles et al. (2020) indicated that not all facets of cognitive flexibility are adversely affected in adults with AN. In adolescents, there was no evidence of deficits in cognitive flexibility compared to healthy groups, a finding that is somewhat consistent with the results reported here. Although intertemporal and food choice studies largely reported increased control through attenuated delay discounting and reduced choice of “tasty-unhealthy” food items, the four studies that did not find group differences in delay discounting were conducted in relatively older or younger samples. Given the preliminary evidence from this review and in others, it is possible that control and rigidity are not necessarily etiological factors in AN, but rather maintaining factors that are developed over time as the illness becomes entrenched.

Habitual responding has also been associated with rigidity that can contribute to compulsive behavior and is believed to contribute to the pathogenesis of AN (Walsh, 2013). Relatedly, domain-general reductions in goal-directed learning are observed in individuals with AN (Foerde et al., 2021), consistent with prior work reporting that attenuation in this learning sytem is broadly linked to compulsivity (Gillan et al., 2016; Voon et al., 2015). This is, perhaps, also reflected in deficiencies in feedback and associative learning that have been reported here, where difficulties incorporating feedback during choice or action selection may be indicative of inflexible responding that is insensitive to change. This may also be the result of very precise expectations or prior beliefs that are built through repetition and not easily altered, but also maintained through avoidance of disconfirming evidence. However, it should be noted that Godier et al. (2016) did not report differences in habitual control during a slips of action test.

### 9.7. Clinical implications

Value-based decision-making during acute illness appears to be impacted in both domain-general (e.g., monetary reward) and domain-specific (e.g., food reward) contexts, resulting in choice behavior that is influenced by some general aversion to uncertainty/risk and broad sensitivity to punishment. Consequently, treatments that work to reduce punishment sensitivity and improve flexible choice behavior in the context of uncertainty or risk may improve the efficacy of psychosocial interventions (Abber et al., 2024; Haynos et al., 2024). Choice behavior in this population also seems to be supported by alterations in neural functioning, with frontostriatal circuitry reflecting a potentially important target for improving decision-making processes. Notably, preliminary findings suggest that high frequency repetitive transcranial magnetic stimulation (HF-rTMS) to the right dlPFC was associated with increased high-fat food choices among inpatients with AN (Muratore et al., 2021). Yet another important consideration is the role of altered latent decision-making processes that may persist even into recovered stages. Many of the studies that reported on R-AN samples observed similar choice behavior to HC groups, however those that included computational methodology to estimate unobserved decision-making subprocesses (e.g., learning rate) noted differences in how choices were made. This may consequently impact trajectories of recovery or even perhaps likelihoods of relapse if these neurocognitive subprocesses are not remediated. These hypotheses notwithstanding, measurement, conceptualization, and treatment of latent processes impacting decision-making behavior may lead to improved outcomes in this population (Haynos et al., 2020).

## 10. Limitations and future directions

One of the primary weaknesses of the existing research has to do with the limited number of prospective or longitudinal studies of value-based decision-making in individuals with AN, limiting inferences that can be made regarding temporal precedence and the etiological or maintaining role of value-based decision-making in AN. Nevertheless, this review is the first step in characterizing differences across illness states to inform future studies. Additionally, more research is needed in adolescent samples, AN subtypes, and in male participants. Indeed, the majority of the studies included here recruited only female participants. While AN tends to have higher prevalence rates in female populations, research examining these processes in males with AN would increase generalizability and contribute to collective understandings of sex-related effects.

Beyond the lack of consistency in operationalization of illness stages across studies, there are also limitations in the metrics used to assess weight-restoration and remittance. For example, the use of BMI as a primary measure of illness stage or outcome has been criticized for being an outdated and inaccurate measure of weight status that does not account for nutritional status or demographic factors (age, sex, race, ethnicity). Assessing body composition or weight suppression may provide more accurate and personalized indicators of physiological health (Gorrell et al., 2019). Additionally, although age is often used to classify adolescent and adult populations, pubertal status is considered a better indicator of developmental stage, and has been hypothesized to be strongly linked to eating disorder development (Klump, 2013). Lastly, the commonly used case-control design of most studies overlooks direct associations between decision-making performance and measures of eating disorder severity.

Future work should also ideally incorporate the constructs proposed in this review to develop more nuanced understandings of decision-making in individuals with AN, as well as implement more longitudinal work to ascertain how executive functioning evolves as a function of age and illness stage and is associated with clinical variables and symptoms of AN. As decisions can have a tremendous impact on the course of one’s illness, whether that encompasses choices about foods, life goals, or money, it is imperative that new developments in task design and methodology be considered in future investigations. Specifically, there is some precedence for studying not only what decisions are made in a particular context, but also how those decisions are made. This can include the valuation process, which is propelled by specific computations that incorporate information about saliency, reward/punishment, risk/uncertainty, habit/goal-direceted systems, and motivation. It is also possible to investigate how valuation is incorporated into decisions about choices, including costs/benefits or the desire to explore choices or exploit what is known about them. These latent behavioral processes can be directly investigated using advanced modeling techniques built to mirror brain-based computations (e.g., drift diffusion modeling) and represent a promising avenue of work that may have considerable clinical implications (Haynos et al., 2022; Scholl and Klein-Flügge, 2018).

Finally, a major limitation is the lack of meta-analyses for tasks that have larger numbers of included studies (e.g., IGT). Meta-analyses in decision-making have been conducted semi-recently for studies using more standard neurocogntiive tasks (Guillaume et al., 2015; Wu et al., 2016), and studies published since the publication of these meta-analyses report on heterogeneous task measures (e.g., studies using the IGT report on net scores, number of advantageous deck selections, computational model parameters), precluding updates to these meta-analyses; however, as more studies in decision-making are published in samples of individuals with AN, future research should conduct updated meta-analyses to add to collective understanding of this executive function in this population.

## 11. Conclusions

Altogether, there is initial evidence that value-based decision-making in both domain-general (e.g., gambling) and disorder-specific (e.g., food choice) contexts may be implicated in AN, particularly during acute illness stages. Decision-making processes could therefore be implicated in the maintenance and progression of the disorder, as maladaptive choices about dietary intake, exercise or engagement in treatment are central in the phenotypic expression of AN. Although differences in raw choice behavior are less pronounced in recovered samples, computational modeling and neuroimaging have revealed altered latent cognitive processes and neural functioning in individuals with AN, providing greater precision than traditional neuropsychological work and suggesting an important avenue of future research. Further, several additonal factors, including reward/punishment, uncertainty/risk, and inflexibility/control appear to disparately influence decision-making and choice behavior and require further research to ascertain their contribution to individual differences in executive functioning within this population. Future work should continue to incorporate longitudinal designs, disorder-specific stimuli, and novel task design and methodology to develop a more precise and idiographic understanding of not just what decisions are made, but how they are made during the course of AN. Such insights may help to reveal how it is that individuals with this disorder can show such persistently dangerous behavior, despite serious and fatal consequences.

## Figures and Tables

**Table 1:** Study Characteristics for Gambling Tasks

*Study*	*Task*	*Subject (n)*	*Sample Type*	*Age (years) Mean (SD)*	*BMI Mean (SD)*	*Stage of Illness (AN only)*	*Female (%)*	*Duration of Illness (years) Mean (SD)*	*Study Type*	*Brain Imaging*	*Quality Assessment Average Score (%)*
Abbate-Daga et al., 2011	Iowa Gambling	HC (30) AN-R (30)	Undefined	24.7 (2.6) 24.1 (6.2)	21.0 (2.2) 15.6 (1.7)	Acute	100% 100%	n/a 5.2 (4.2)	Cross-Sectional	No	A (85.0)
Adoue et al., 2015	Iowa Gambling	HC (49) AN (63)	Adult	30.3 (11.2) 24.8 (7.1)	21.3 (2.7) 16.1 (1.6)	Acute	100% 100%	n/a 6.2 (7.1)	Cross-Sectional	No	B (76.2)
Aloi et al., 2015	Iowa Gambling	HC (45) AN (45) BED (45)	Undefined	25.6 (3.5) 22.8 (5.6) 30.6 (10.9)	20.2 (1.6) 15.5 (1.4) 35.2 (6.5)	Acute	100% 100% 100%	n/a No Data No Data	Cross-Sectional	No	A (81.0)
Bodell et al., 2014	Iowa Gambling	HC (20) AN (22)	Undefined	No Data 25.6 (5.8)	No Data 15.8 (2.3) 20.6 (0.9)^1^	Acute & Weight-Restored^1^	100% 100%	n/a 5.9 (5.5)	Longitudinal	Yes	A (86.4)
Bosanac et al., 2007	Iowa Gambling	HC (16) AN (16) W-AN (12) BN (13)	Undefined	23.8 (6.1) 28.9 (9.6) 28.9 (7.7) 28.3 (9.1)	22.3 (2.8) 15.2 (1.4) 20.5 (2.1) 23.5 (5.5)	Acute & Weight-Restored	100% 100% 100% 100%	n/a 12.9 (10.4) 9.7 (6.0) 10.5 (9.1)	Cross-Sectional	No	B (70.0)
Brogan et al., 2010	Iowa Gambling	HC (20) AN (22) BN (17) OB (18)	Adult	27.8 (7.0) 29.1 (7.4) 29.9 (6.4) 52.1 (11.7)	21.6 (1.4) 16.0 (2.0) 31.9 (9.4) 36.2 (5.0)	Acute	100% 100% 100% 100%	n/a No Data No Data n/a	Cross-Sectional	No	A (81.0)
Cavedini et al., 2004	Iowa Gambling	HC (82) AN-R (26) AN-BP (33)	Undefined	30.9 (10.7) 21.7 (3.2) 23.4 (4.4)	No Data 13.5 (1.5) 15.6 (2.2)	Acute	52.4% 96.1% 96.9%	n/a 4.1 (2.9) 6.3 (3.6)	Cross-Sectional	No	B (75.0)
Cavedini et al., 2006	Iowa Gambling	HC (30) AN-R (18) AN-BP (20)	Undefined	22.6 (4.1) 23.8 (4.4) 21.5 (3.5)	No Data 13.2 (1.3) 14.9 (1.8)^1^ 15.1 (1.6) 16.8 (1.9)^1^	Acute & Discharge^1^	100% 100% 100%	n/a 3.3 (2.6) 6.9 (3.5)	Longitudinal	No	B (76.2)
Chan et al., 2014	Iowa Gambling	HC (67) AN (94) BN (63)	Undefined	25.5 (6.7) 25.6 (8.5) 26.9 (10.8)	20.6 (1.8) 15.5 (1.9) 22.8 (4.6)	Acute	No Data 93.6% 98.4%	n/a No Data No Data	Cross-Sectional	Yes	A (85.7)
Danner et al., 2012	Iowa Gambling	HC (15) AN (16) R-AN (15)	Undefined	25.8 (4.7) 25.6 (5.4) 24.3 (4.7)	21.5 (2.3) 14.7 (1.7) 21.2 (1.8)	Acute & Remitted	100% 100% 100%	n/a 7.9 (4.0) 4.0 (2.7)	Cross-Sectional	No	A (85.7)
Danner et al., 2016	Iowa Gambling	HC (40) AN-R (32) AN-BP (32)	Undefined	24.4 (5.8) 23.2 (6.6) 27.2 (9.3)	21.7 (2.6) 17.3 (1.7) 17.1 (1.9)	Acute	100% 100% 100%	n/a 5.7 (4.9) 10.0 (9.9)	Cross-Sectional	No	A (85.7)
Di Lodovico et al., 2022	Iowa Gambling	HC (204) AN (91) AN-UR (90) R-AN (23)	Undefined	37.6 (15.7) 27.5 (7.3) 52.1 (11.5) 29.5 (4.5)	22.4 (3.3) 16.1 (1.6) 22.8 (3.9) 20.2 (1.2)	Acute & Remitted	100% 100% 100% 100%	n/a 7.3 (6.6) n/a 5.3 (4.0)	Cross-Sectional	No	A (85.7)
Elzakkers et al., 2016	Iowa Gambling	AN (70)	Adult	27.3 (9.7)	15.5 (1.9)	Acute	100%	8.6 (8.1)	Cross-Sectional	No	B (57.1)
Fagundo et al., 2012	Iowa Gambling	HC (137) AN (35) OB (52)	Adult	24.8 (7.0) 28.1 (8.2) 40.5 (11.1)	21.5 (2.7) 17.2 (1.4) 39.8 (7.4)	Acute	100% 100% 100%	n/a No Data n/a	Cross-Sectional	No	B (65.0)
Fornasari et al., 2014	Iowa Gambling	HC (15) AN (15)	Adolescent	15.4 (1.2) 15.5 (1.3)	20.6 (2.0) 17.0 (1.1)	Acute	100% 100%	n/a 1.1^2^ (No Data)	Cross-Sectional	No	B (70.0)
Galimberti et al., 2013	Iowa Gambling	HC (29) HC-R (29) AN (29) AN-UR (29)	Adult	28.6 (11.9) 43.3 (13.6) 24.1 (6.8) 43.8 (12.9)	No Data No Data 16.2 (4.02) No Data	Acute	100% 100% 100% 100%	n/a n/a 6.0 (5.1) n/a	Cross-Sectional	No	A (85.0)
Garrido & Subirá, 2013	Iowa Gambling	HC (38) AN-R (27) BP-G (44)	Undefined	23.3 (4.6) 25.9 (7.9) 28.2 (8.1)	No Data 15.6 (2.1) 20.0 (4.0)	Acute	100% 100% 100%	n/a 6.9 (4.6) 8.9 (8.0)	Cross-Sectional	No	B (66.7)
Giannunzio et al., 2018	Iowa Gambling	HC (301) AN (310)	Mixed	16.5 (1.3)^3^ 26.8 (5.5) 16.8 (1.2)^3^ 26.2 (6.6)	21.0 (2.4)^3^ 21.5 (2.9) 17.1 (2.5)^3^ 18.1 (3.0)	Acute & Weight-Restored	100% 100%	n/a 1.4 (1.1)^3^ 5.6 (5.6)	Cross-Sectional	No	A (85.0)
Guillaume et al., 2010	Iowa Gambling	HC (83) AN (49) BN (38)	Adult	28.0^2^ (No Data) 23.3^2^ (No Data) 23.0^2^ (No Data)	20.2^2^ (No Data) 15.4^2^ (No Data) 21.3^2^ (No Data)	Acute	100% 100% 100%	n/a 3.0^2^ (No Data) 5.0^2^ (No Data)	Cross-Sectional	No	B (63.2)
Jollant et al., 2007	Iowa Gambling	AN (39) BN (32) SUD (64) AD (125) OCD (20) BD (66) UD (199)	Adult	32.9 (No Data)^2,4^	No Data	Acute	74.1%^4^	No Data	Cross-Sectional	No	B (76.2)
Lindner et al., 2012	Iowa Gambling	HC (100) R-AN (100)	Undefined	34.5 (7.3) 34.4 (7.1)	21.8 (1.4) 20.9 (1.3)	Remitted	100% 100%	n/a 3.9 (3.2)	Cross-Sectional	No	B (57.1)
Matsumoto et al., 2015	Iowa Gambling	HC (51) AN (22) BN (36)	Adult	23.8 (5.6) 25.8 (6.3) 25.9 (5.8)	21.0 (1.7) 15.9 (2.6) 19.8 (2.4)	Acute	100% 100% 100%	n/a 7.2 (6.5) 7.2 (5.8)	Cross-Sectional	No	A (85.7)
Matsumoto et al., 2017	Iowa Gambling	HC (22) AN (19) BN (28)	Undefined	24.9 (6.6) 24.4 (7.7) 26.2 (5.7)	20.8 (1.7) 14.5 (1.8) 19.8 (2.7)	Acute	100% 100% 100%	n/a 6.2 (6.5) 7.7 (6.1)	Cross-Sectional	No	B (65.0)
Miranda-Olivos et al., 2021	Iowa Gambling	HC (123) AN-R (59) AN-BP (27) BED (30)	Adult	26.2 (7.9) 26.4 (8.5) 28.7 (8.9) 35.6 (10.3)	21.7 (2.7) 16.1 (1.5) 16.7 (2.0) 30.8 (9.2)	Acute	100% 100% 100% 100%	n/a 5.2 (6.1) 8.4 (4.9) 10.0 (7.8)	Cross-Sectional	No	A (90.0)
Neveu et al., 2016	Gambling	HC (18) AN-R (16) AN-BP (19) BN (18)	Adult	24.3 (3.2) 21.7 (5.0) 25.6 (4.9) 24.2 (5.8)	21.5 (3.0) 15.3 (1.2) 17.6 (1.8) 20.6 (3.2)	Acute	100% 100% 100% 100%	n/a No Data No Data No Data	Cross-Sectional	No	B (52.4)
Paslakis et al., 2019	Iowa Gambling	HC (106) AN (51)	Adult	27.2 (7.8) 27.4 (8.7)	21.7 (2.9) 17.4 (1.6)	Acute	100% 100%	n/a 7.3 (6.3)	Cross-Sectional	No	B (80.0)
Perpiñá et al., 2017	Iowa Gambling	HC (39) AN-R (18) EDNOS-AN (21) BP-G (47) OB (27)	Mixed	31.9 (13.5) 22.3 (7.6) 23.1 (8.7) 24.5 (7.4) 47.8 (11.5)	23.2 (3.5) 15.9 (1.2) 19.1 (1.3) 21.3 (3.6) 43.9 (10.0)	Acute	76.9% 100% 90.5% 95.7% 85.2%	n/a No Data No Data No Data No Data	Cross-Sectional	No	A (81.0)
Steward et al., 2016	Iowa Gambling	HC (46) AN (42)	Adult	28.2 (7.2) 28.8 (9.7)	21.0 (No Data) 21.4 (No Data)^1^ 16.4 (No Data)^5^ 18.8 (No Data)^1,5^ 16.7 (No Data)^6^ 19.1 (No Data)^1,6^	Acute & Partially Remitted^1,5^ & Remitted^1,6^	100% 100%	n/a No Data	Longitudinal	No	B (71.4)
Steward et al., 2019	Iowa Gambling	HC (51) AN (51)	Adult	26.8 (7.3) 27.4 (8.7)	21.4 (1.8) 17.1 (1.3)	Acute	100% 100%	n/a No Data	Cross-Sectional	No	B (71.4)
Tchanturia et al., 2007	Iowa Gambling	HC (29) AN (29) R-AN (14)	Undefined	26.3 (7.9) 28.5 (9.2) 28.9 (7.4)	22.1 (2.4) 15.5 (1.3) 20.3 (1.9)	Acute & Remitted	100% 100% 100%	n/a 8.2 (5.2) 5.0 (3.4)	Cross-Sectional	No	A (85.7)
Tchanturia et al., 2012	Iowa Gambling	HC (61) AN (48)	Undefined	22.2 (5.7)^7^ 25.5 (7.6)^8^ 27.5 (7.5)^7^ 27.2 (8.5)^8^	22.1 (3.9)^7^ 23.5 (3.8)^8^ 16.6 (1.2)^7^ 17.5 (2.6)^8^	Acute	67.2% 60.4%	n/a 9.1 (5.7)^7^ 7.3 (6.5)^8^	Cross-Sectional	No	B (80.0)
Tenconi et al., 2016	Iowa Gambling	HC (98) AN (91)^9^	Mixed	23.5 (6.3) 22.6 (6.9)	21.6 (3.3) 17.6 (3.0)	Acute	100% 100%	n/a No Data	Cross-Sectional	No	B (71.4)
Tenconi et al., 2021	Iowa Gambling	HC (58) AN (56)	Mixed	19.5 (4.9) 19.6 (5.2)	21.3 (2.2) 15.6 (1.5)	Acute	100% 100%	n/a 2.0 (2.2)	Longitudinal	No	B (66.7)
Testa et al., 2021	Iowa Gambling	HC (171) AN-R (107) GD (121) OB -T2D (115) OB +T2D (67)	Undefined	29.7 (13.3) 25.3 (8.3) 38.3 (13.6) 41.4 (11.9) 54.6 (11.3)	22.3 (3.1) 16.4 (2.2) 26.4 (5.9) 44.6 (6.7) 42.0 (8.6)	Acute	84.2% 90.7% 15.7% 93% 71.6%	n/a No Data No Data No Data No Data	Cross-Sectional	No	B (75.0)
Verharen et al., 2019	Iowa Gambling	HC (55) AN (60) AN (216)^10^	Adult	24.5 (8.3) 27.3 (9.9) 22.3 (7.3)^10^	21.7 (2.8) 15.4 (1.9) 16.4 (2.4)^10^	Acute	100% 100% 96%^10^	n/a 8.6 (8.1) No Data	Cross-Sectional	No	B (72.7)
Yokokura et al., 2019	Iowa Gambling	HC (20) AN-R (12) AN-BP (10)	Adult	22.4 (3.2) 24.3 (6.0) 25.4 (6.2)	20.5 (2.2) 13.8 (1.3) 15.2 (1.3)	Acute	100% 100% 100%	n/a 4.2 (5.0) 6.4 (6.5)	Cross-Sectional	Yes	B (75.0)

*Note*. Although this review focuses on anorexia nervosa, for comprehensiveness, this table includes all comparison groups included in each study. AN – anorexia nervosa; R-AN – recovered anorexia nervosa; AN-R – restricting subtype anorexia nervosa; AN-BP – binge/purge subtype anorexia nervosa; AN-UR – anorexia nervosa unaffected relatives; BN – bulimia nervosa; BED – binge eating disorder; OB – obesity; EDNOS-AN – eating disorder not otherwise specified, anorexia nervosa type; BP-G – binge/purge group; SUD – substance use disorder; AD – anxiety disorder; OCD – obsessive-compulsive disorder; BD – bipolar disorder; UD – unipolar disorder; HC – healthy control; GD – gambling disorder; HC-R – healthy control relatives.

1 Denotes values at time point 2

2 Denotes median values; mean values were not reported

3 Denotes values for adolescent sample

4 Denotes values calculated across all sample groups

5 Denotes values for partially remitted sample

6 Denotes values for fully remitted sample

7 Denotes values for female sample

8 Denotes values for male sample

9 33 anorexia nervosa participants were weight-restored, defined as BMI ≥ 18

10 Denotes values for second cohort of AN sample

**Table 2:** Study Characteristics for Intertemporal Choice Tasks

*Study*	*Task*	*Subject (n)*	*Sample Type*	*Age (years) Mean (SD)*	*BMI Mean (SD)*	*Stage of Illness (AN only)*	*Female (%)*	*Duration of Illness (years) Mean (SD)*	*Study Type*	*Brain Imaging*	*Quality Assessment Average Score (%)*
Bartholdy et al., 2017	Delay Discounting	HC (28) AN (28) BN (27) BED (11)	Undefined	24.6 (5.1) 30.0 (10.5) 25.3 (6.9) 28.7 (11.3)	22.0 (2.0) 16.6 (1.8) 22.6 (3.3) 28.9 (6.9)	Acute	100% 100% 100% 100%	n/a 10.6 (10.0) 5.0 (5.5) 2.3 (4.0)	Cross-Sectional	No	B (80.0)
Bernardoni et al., 2020	Delay Discounting	HC (119) AN-R (94) R-AN-R (37)	Mixed	18.6 (4.5) 16.1 (3.1) 22.2 (3.8)	20.9 (2.2) 14.6 (1.4) 20.6 (1.6)	Acute & Remitted	100% 100% 100%	n/a No Data No Data	Cross-Sectional	No	A (90.5)
Bernardoni et al., 2023	Delay Discounting	HC (55) AN (55)	Mixed	16.1 (2.0) 16.0 (2.0)	20.8 (2.2) 14.8 (1.5)	Acute	100% 100%	n/a No Data	Cross-Sectional	No	A (81.0)
Decker et al., 2015	Delay Discounting	HC (39) AN (59)	Mixed	24.7 (7.6) 20.7 (2.8)^1^ 25.0 (7.5) 19.3 (2.5)^1^	21.7 (1.9) 21.4 (1.8)^1^ 16.6 (1.5) 16.8 (1.4)^1^	Acute & Weight-Restored	94.9% 100%^1^ 96.6% 100%^1^	n/a No Data	Longitudinal	Yes	A (81.8)
Doose et al., 2020	Delay Discounting	AN (22)	Adolescent	15.5 (2.2) 15.8 (2.2)^2^	14.9 (1.2) 18.8 (1.2)^2^	Acute & Partially Weight-Restored^2^	100%	No Data	Longitudinal	Yes	A (81.8)
King et al., 2016	Intertemporal Choice	HC (31) AN (31)	Mixed^3^	16.1 (2.4) 15.7 (2.5)	20.4 (2.0) 14.7 (1.3)	Acute	100% 100%	n/a No Data	Cross-Sectional	Yes	B (76.2)
King et al., 2020	Intertemporal Choice	HC (36) R-AN (36)	Mixed	21.2 (3.4) 22.2 (3.3)	21.6 (1.8) 21.1 (1.9)	Remitted	100% 100%	n/a 2.9 (2.0)	Cross-Sectional	Yes	A (81.8)
Ritschel et al., 2015	Intertemporal Choice	HC (54) AN (34) W-AN (33)	Mixed	18.8 (4.4) 15.3 (2.7) 21.7 (3.1)	21.1 (2.9) 14.7 (1.3) 21.0 (1.9)	Acute & Weight-Restored	100% 100% 100%	n/a 2.3 (2.1) No Data	Longitudinal	No	A (90.5)
Steinglass et al., 2012	Intertemporal Choice	HC (28) AN (36)	Undefined	25.9 (6.7) 24.8 (6.4)	21.5 (2.4) 16.3 (1.6)	Acute	100% 100%	n/a No Data	Cross-Sectional	No	A (85.7)
Steinglass et al., 2017	Intertemporal Choice; Titration	HC (75) AN (27) OCD (50) SAD (44)	Adult	29.0 (7.6) 27.7 (7.5) 29.2 (5.8) 30.0 (4.0)	24.1 (4.4) 17.5 (1.0) 24.6 (5.3) 23.9 (6.3)	Acute	52% 100% 48% 57%	n/a No Data No Data No Data	Cross-Sectional	No	A (81.8)
Stern et al., 2024	Delay Discounting	HC (19) AN-R (28) ARFID (57)	Mixed	21.3 (7.6) 20.8 (3.8) 17.4 (5.4)	28.1 (10.5) 18.5 (2.2) 24.0 (6.1)	Acute	37% 60% 100%	n/a No Data No Data	Cross-Sectional	No	A (90.0)
Steward et al., 2017	Delay Discounting	HC (80) AN-R (37) AN-BP (19) BED (24)	Adult	23.0 (4.4) 24.3 (7.2) 28.6 (6.6) 33.6 (8.6)	21.6 (3.2) 16.2 (1.8) 16.7 (0.9) 38.9 (9.7)	Acute	100% 100% 100% 100%	n/a 5.4 (6.0) 10.5 (8.9) 12.8 (8.8)	Cross-Sectional	No	B (80.0)
Wierenga et al., 2015	Delay Discounting	HC (17) R-AN (23)	Adult	25.3 (1.4) 27.7 (1.6)	22.6 (0.7) 21.6 (0.3)	Remitted	100% 100%	n/a No Data	Cross-Sectional	Yes	A (85.0)

*Note*. Although this review focuses on anorexia nervosa, for comprehensiveness, this table includes all comparison groups included in each study. AN – anorexia nervosa; W-AN – weight-recovered anorexia nervosa; R-AN – recovered anorexia nervosa; AN-R – restricting subtype anorexia nervosa; R-AN-R – recovered restricting subtype anorexia nervosa; AN-BP – binge/purge subtype anorexia nervosa; BED – binge eating disorder; OCD – obsessive-compulsive disorder; SAD – social anxiety disorder; BN – bulimia nervosa; ARFID – avoidant/restrictive food intake disorder; HC – healthy control/non-clinical.

1 Denotes subjects that participated in fMRI portion of study

2 Denotes values at time point 2

3 Predominately adolescent sample

**Table 3:** Study Characteristics for Food Choice Tasks

*Study*	*Task*	*Subject (n)*	*Sample Type*	*Age (years) Mean (SD)*	*BMI Mean (SD)*	*Stage of Illness (AN only)*	*Female (%)*	*Duration of Illness (years) Mean (SD)*	*Study Type*	*Brain Imaging*	*Quality Assessment Average Score (%)*
Cowdrey et al., 2013	Food Choice	HC (41) AN (20) R-AN (22) W-AN (22)	Mixed	24.3 (6.5) 26.4 (10.6) 23.7 (5.8) 25.1 (6.0)	21.7 (1.9) 16.3 (1.1) 21.0 (1.5) 21.1 (1.9)	Acute & Weight-Restored & Remitted	100% 100% 100% 100%	n/a No Data No Data No Data	Cross-Sectional	No	A (81.8)
Foerde et al., 2015	Food Choice	HC (21) AN (21)	Undefined	22.7 (3.1) 26.1 (6.5)	21.5 (1.9) 15.7 (2.0)	Acute	100% 100%	n/a 11.4 (6.7)	Cross-Sectional	Yes	A (81.8)
Foerde et al., 2021	Food Choice	HC (29) AN (24)	Adult	25.8 (5.2) 26.9 (6.5)	21.0 (1.4) 21.0 (1.6)^1^ 16.3 (1.9) 20.5 (0.9)^1^	Acute & Weight-Restored^1^	100% 100%	n/a 8.6 (6.9)	Longitudinal	Yes	A (81.8)
Foerde et al., 2022	Food Choice	HC (36) AN (35) S-AN (19) HC-D (20)	Adult	25.8 (5.3) 26.7 (6.6) 23.5 (5.9) 26.0 (5.4)	21.0 (1.6) 16.1 (1.7) 20.9 (1.6) 22.1 (1.5)	Acute	100% 100% 100% 100%	n/a 9.7 (7.1) 5.9 (5.9) n/a	Cross-Sectional	Yes	A (81.8)
Steinglass et al., 2015	Food Choice	HC (20) AN (22)	Mixed	26.3 (5.8) 29.4 (11.2)	21.0 (1.7) 17.5 (1.9)	Acute	95% 95.2%	n/a No Data	Cross-Sectional	No	A (81.0)
Uniacke et al., 2020	Food Choice	AN-R (40) AN-BP (46)	Mixed	27.8 (7.7) 26.4 (6.3)	15.6 (2.1) 16.9 (1.7)	Acute	100% 100%	10.9 (7.8) 10.4 (6.2)	Cross-Sectional	Yes	A (85.0)
Xue et al., 2022	Food Choice	HC (21) AN (21)	Mixed	22.7 (3.1) 26.4 (6.5)	21.5 (1.9) 15.7 (2.1)	Acute	100% 100%	n/a No Data	Cross-Sectional	Yes	A (81.8)

*Note*. Although this review focuses on anorexia nervosa, for comprehensiveness, this table includes all comparison groups included in each study. AN – anorexia nervosa; AN-R – anorexia nervosa restricting subtype; AN-BP – anorexia nervosa binge/purge subtype; S-AN – treatment-seeking women with clinically significant restrictive eating and BMI of 18.5–25.0; HC – healthy control; HC-D – healthy control with self-reported restrictive eating behavior that led to weight loss.

1 Denotes values at time point 2

**Table 4:** Study Characteristics for Balloon Analogue, Beads, and Wheel of Fortune Tasks

*Study*	*Task*	*Subject (n)*	*Sample Type*	*Age (years) Mean (SD)*	*BMI Mean (SD)*	*Stage of Illness (AN only)*	*Female (%)*	*Duration of Illness (years) Mean (SD)*	*Study Type*	*Brain Imaging*	*Quality Assessment Average Score (%)*
Adoue et al., 2015	Balloon Analogue Risk	HC (49) AN (63)	Adult	30.3 (11.2) 24.8 (7.1)	21.3 (2.7) 16.1 (1.6)	Acute	100% 100%	n/a 6.2 (7.1)	Cross-Sectional	No	B (76.2)
Jenkinson et al., 2023	Balloon Analogue Risk	HC-L (38)^1^ HC-H (35)^1^ AN (31)^1^ R-AN (23)^1^	Adult	22.9 (3.3) 22.5 (4.7) 24.9 (8.7) 26.1 (7.5)	19.5 (0.9) 19.7 (0.8) 15.9 (1.5) 19.7 (1.7)	Acute & Remitted	100% 100% 100% 100%	n/a n/a 7.6 (8.2) 4.7 (4.5)	Cross-sectional	No	A (90.9)
McKenna et al., 2014	Beads	HC (33) AN (27)	Adult	27.0 (9.1) 32.0 (13.5)	22.5 (3.1) 15.6 (1.1)	Acute	93.9% 92.9%	n/a 9.1 (11.3)^2^	Cross-Sectional	No	A (81.8)
Neveu et al., 2016	Balloon Analogue Risk Gambling	HC (18) AN-R (16) AN-BP (19) BN (18)	Adult	24.3 (3.2) 21.7 (5.0) 25.6 (4.9) 24.2 (5.8)	21.5 (3.0) 15.3 (1.2) 17.6 (1.8) 20.6 (3.2)	Acute	100% 100% 100% 100%	n/a No Data No Data No Data	Cross-Sectional	No	B (52.4)
Piccolo et al., 2019a	Wheel of Fortune	HC (17) AN (24)	Mixed	23.0 (No Data) 23.0 (No Data)	21.8 (No Data) 14.4 (No Data)	Acute	100% 100%	n/a No Data	Cross-sectional	No	A (85.7)
Piccolo et al., 2019b	Wheel of Fortune	AN (12)	Mixed	22.2 (1.3) 22.2 (1.3)^3^	14.6 (0.2) 18.1 (0.2)^3^	Acute & Weight-Restored^3^	100%	No Data	Longitudinal	No	B (71.4)
Sternheim et al., 2011	Beads	HC (39) AN (37) BN (22)	Mixed	27.5 (10.1) 25.6 (8.5) 26.1 (5.6)	21.5 (2.2) 16.4 (1.3) 22.7 (4.2)	Acute	100% 100% 100%	n/a No Data No Data	Cross-Sectional	No	B (76.2)
Wittorf et al., 2012	Beads	HC (55) AN (15) Schizophrenia (20) MDD (20)	Adult	31.7 (10.6) 23.9 (5.7) 35.3 (9.0) 36.3 (9.7)	No Data 15.8 (1.9) No Data No Data	Acute	61.8% 100% 35% 60%	n/a No Data No Data No Data	Cross-Sectional	No	A (85.7)

*Note*. Although this review focuses on anorexia nervosa, for comprehensiveness, this table includes all comparison groups included in each study. AN – anorexia nervosa; AN-R – anorexia nervosa restricting subtype; AN-BP – anorexia nervosa binge/purge subtype; R-AN – recovered anorexia nervosa; BN – bulimia nervosa; MDD – major depressive disorder; HC – healthy control/non-clinical; HC-L – healthy control characterized by low disordered eating; HC-H – healthy control characterized by high disordered eating.

1 Sample described here is from the clinical study only; no results reported from the non-clinical study

2 Time diagnosed

3 Denotes values at time point 2

**Table 5:** Study Characteristics for Probabilistic Reversal Learning Tasks

*Study*	*Task*	*Subject (n)*	*Sample Type*	*Age (years) Mean (SD)*	*BMI Mean (SD)*	*Stage of Illness (AN only)*	*Female (%)*	*Duration of Illness (years) Mean (SD)*	*Study Type*	*Brain Imaging*	*Quality Assessment Average Score (%)*
Adoue et al., 2015	Probabilistic Reversal Learning	HC (49) AN (63)	Adult	30.3 (11.2) 24.8 (7.1)	21.3 (2.7) 16.1 (1.6)	Acute	100% 100%	n/a 6.2 (7.1)	Cross-Sectional	No	B (76.2)
Bernardoni et al., 2017	Probabilistic Reversal Learning	HC (36) AN (36)	Mixed	16.3 (2.6) 16.0 (2.6)	20.4 (2.5) 14.7 (1.3)	Acute	100% 100%	n/a No Data	Cross-Sectional	Yes	A (86.4)
Bernardoni et al., 2021	Probabilistic Reversal Learning	HC (31) R-AN-R (31)	Mixed	22.0 (2.9) 22.3 (2.8)	21.3 (2.1) 21.0 (1.9)	Remitted	100% 100%	n/a No data	Cross-Sectional	Yes	A (86.4)
Geisler et al., 2017	Probabilistic Reversal Learning	HC (36) AN (36)	Mixed	16.3 (2.6) 16.0 (2.6)	20.4 (2.5) 14.7 (1.3)	Acute	100% 100%	n/a No Data	Cross-Sectional	Yes	A (90.9)
Ritschel et al., 2017	Probabilistic Reversal Learning	HC (31) R-AN-R (31)	Mixed	22.1 (3.0) 22.3(2.8)	21.3 (2.1) 21.0 (1.9)	Remitted	100% 100%	n/a No Data	Cross-Sectional	Yes	A (85.7)
Sarrar et al., 2011	Probabilistic Object Reversal	HC (28) AN (30)	Adolescent	16.3 (1.3) 16.2 (1.2)	20.5 (2.5) 20.6 (2.8)^1^ 15.0 (1.2) 17.4 (0.7)^1^	Acute	100% No Data	n/a No Data	Longitudinal	No	A (90.5)

*Note*. Although this review focuses on anorexia nervosa, for comprehensiveness, this table includes all comparison groups included in each study. AN – anorexia nervosa; R-AN – recovered anorexia nervosa; R-AN-R – recovered restricting subtype anorexia nervosa; HC – healthy control.

1 Denotes values at time point 2

**Table 6:** Study Characteristics for Other Tasks

*Study*	*Task*	*Subject (n)*	*Sample Type*	*Age (years) Mean (SD)*	*BMI Mean (SD)*	*Stage of Illness (AN only)*	*Female (%)*	*Duration of Illness (years) Mean (SD)*	*Study Type*	*Brain Imaging*	*Quality Assessment Average Score (%)*
Bischoff-Grethe et al., 2013	Monetary Guessing	HC (12) AN-R (10)	Adolescent	15.4 (1.6) 16.2 (1.8)	21.5 (1.7) 16.4 (1.4)	Acute	100% 100%	n/a 2.6 (2.2)	Cross-Sectional	Yes	A (86.4)
Foerde et al., 2017	Acquired Equivalence	HC (26) AN (36)	Mixed	22.9 (5.6) 24.7 (8.1)	21.6 (1.7) 16.8 (1.3) 20.3 (0.7)^1^	Acute & Weight-Restored^1^	92.3% 97.2%	n/a 8.1 (7.4)	Longitudinal	No	A (90.5)
Foerde et al., 2021	Two-Step	HC (53) AN (41)	Mixed	25.6 (5.0) 27.1 (7.0)	21.3 (1.5) 20.9 (1.3)^1^ 16.0 (2.0) 20.3 (1.0)^1^	Acute & Weight-Restored^1^	100% 100%	n/a 9.6 (7.1)	Longitudinal	No	B (77.3)
Godier et al., 2016	Slips of Action	HC (18)^2^ AN (23)^2^ HC (17)^3^ AN-R (13)^3^ R-AN (14)^3^	Adults	31.8 (7.5)^2^ 25.7 (6.4)^2^ 24.1 (5.6)^3^ 31.2 (8.0)^3^ 27.1 (6.5)^3^	24.4 (2.8)^2^ 16.8 (1.9)^2^ 21.2 (1.9)^3^ 15.8 (1.9)^3^ 20.9 (1.6)^3^	Acute & Remitted	100%^2^ 100%^2^ 100%^3^ 100%^3^ 100%^3^	n/a 11.3 (6.9)^2^ n/a 10.3 (5.2)^3^ 5.8 (4.2)^3^	Cross-Sectional	No	A (90.5)
Gu et al., 2024	Self-Referential Processing Task	HC (38) AN (35)	Mixed	23.8 (6.0) 22.4 (6.5)	22.3 (2.1) 19.1 (2.1)	Acute	100% 100%	n/a No Data	Cross-sectional	No	A (86.4)
Isobe et al., 2018	Ultimatum Game	HC (22) AN (24)	Adult	34.6 (9.7) 35.9 (10.0)	21.8 (3.4) 14.3 (2.7)	Acute	100% 100%	n/a 16.7 (9.3)	Cross-Sectional	No	B (60.0)
Pike et al., 2023	Volatility	HC (32) EA (25) R-AN (25)	Adult	25.0 (6.4) 23.8 (5.2) 23.5 (3.8)	23.1 (4.8) 21.8 (2.8) 21.8 (2.4)	Remitted	100% 100% 100%	n/a n/a No Data	Cross-Sectional	No	A (95.5)
Wagner et al., 2007	Monetary Guessing	HC (13) R-AN-R (13)	Undefined	26.4 (6.9) 26.6 (6.8)	22.6 (2.0) 20.7 (2.4)	Remitted	100% 100%	n/a No Data	Cross-Sectional	Yes	B (75.0)
Wierenga et al., 2021	Probabilistic Associative Learning	HC (38) AN-R (42)	Mixed	21.6 (4.3) 22.8 (9.6)	21.7 (2.2) 18.3 (2.2)	Acute & Partially Remitted	100% 100%	n/a No Data	Cross-Sectional	No	A (86.4)

*Note*. Although this review focuses on anorexia nervosa, for comprehensiveness, this table includes all comparison groups included in each study. AN – anorexia nervosa; R-AN – recovered anorexia nervosa; AN-R – restricting subtype anorexia nervosa; R-AN-R – recovered restricting subtype anorexia nervosa; HC – healthy control; EA – high-threshold eating disorder symptoms without diagnosis.

1 Denotes values at time point 2

2 Denotes Study 1 values

3 Denotes Study 2 values
